# The association between environmental exposures to chlordanes, adiposity and diabetes-related features: a systematic review and meta-analysis

**DOI:** 10.1038/s41598-021-93868-4

**Published:** 2021-07-15

**Authors:** Vânia Mendes, Cláudia Ribeiro, Inês Delgado, Bárbara Peleteiro, Martine Aggerbeck, Emilie Distel, Isabella Annesi-Maesano, Denis Sarigiannis, Elisabete Ramos

**Affiliations:** 1 ISPUP-EPIUnit, Institute of Public Health, Universidade do Porto, Rua das Taipas, nº135, 4050-600 Porto, Portugal; 2 Departamento de Ciências da Saúde Pública e Forenses, e Educação Médica da Faculdade de Medicina da Universidade do Porto, Al. Prof. Hernâni Monteiro, 4200-319 Porto, Portugal; 3 INSERM UMR-S 1124, 45 rue des Saints Pères, 75006 Paris, France; 4 Université de Paris, 45 rue des Saints Pères, 75006 Paris, France; 5 Sorbonne Université and INSERM, Epidemiology of Allergic and Respiratory Diseases Department (EPAR), Pierre Louis Institute of Epidemiology and Public Health (IPLESP UMRS 1136), Saint-Antoine Medical School, Paris, France; 6 Department of Chemical Engineering, Aristotle University of Thessaloniki, 54124 Thessaloniki, Greece

**Keywords:** Epidemiology, Endocrine system and metabolic diseases, Diabetes, Obesity

## Abstract

Chlordane compounds (CHLs) are components of technical chlordane listed in the Stockholm convention on persistent organic pollutants identified as endocrine disrupting chemicals (EDCs) and may interfere with hormone biosynthesis, metabolism or action resulting in an unbalanced hormonal function. There is increasing scientific evidence showing EDCs as risk factors in the pathogenesis and development of obesity and obesity-related metabolic syndromes such as type 2 diabetes, but there is no systematized information on the effect of CHLs in humans. Our aim is to identify the epidemiological data on the association between CHLs with adiposity and diabetes using a systematic approach to identify the available data and summarizing the results through meta-analysis. We searched PubMed and Web of Science from inception up to 15 February 2021, to retrieve original data on the association between chlordanes, and adiposity or diabetes. For adiposity, regression coefficients and Pearson or Spearman correlation coefficients were extracted and converted into standardized regression coefficients. Data were combined using fixed effects meta-analyses to compute summary regression coefficients and corresponding 95% confidence intervals (95% CI). For the association between chlordanes and diabetes, Odds ratios (ORs) were extracted and the DerSimonian and Laird method was used to compute summary estimates and respective 95% CI. For both, adjusted estimates were preferred, whenever available. Among 31 eligible studies, mostly using a cross-sectional approach, the meta-analysis for adiposity was possible only for oxychlordane and transchlordane, none of them were significantly associated with adiposity [(β = 0.04, 95% CI 0.00; 0.07, I^2^ = 89.7%)] and (β = 0.02, 95% CI − 0.01; 0.06), respectively. For diabetes, the estimates were positive for all compounds but statistically significant for oxychlordane [OR = 1.96 (95% CI 1.19; 3.23)]; for trans-nonachlor [OR = 2.43 (95% CI 1.64; 3.62)] and for heptachlor epoxide [OR = 1.88 (95% CI 1.42; 2.49)]. Our results support that among adults, the odds of having diabetes significantly increase with increasing levels of chlordanes. The data did not allow to reach a clear conclusion regarding the association with adiposity.

## Introduction

Environmental chemical exposures have gained great attention in the past decades based on the growing awareness on the role that the environment plays in health. According to a systematic review performed in 2011, the global burden of disease accountable to environmental exposures and management of selected chemicals amounts to at least 4.9 million deaths per year, representing 8.3% of mortality and 5.7% of the total burden of disease in disability-adjusted life years (DALYs) worldwide^1^. Furthermore, it is expected that the total environmental burden of disease is underestimated^2^.

Chlordane compounds (CHLs) are components of technical chlordane listed in the Stockholm Convention on Persistent Organic Pollutants^3^. The major components are trans-chlordane (13%), cis-chlordane (11%), trans-nonachlor (5%) and heptachlor (5%) but more thanh 30 less abundant chemicals were also identified^4^. In humans, the exposure occurs primarily through food intake, but also by inhalation or skin contact^5^.

Chlordane is a synthetic organochlorine pesticide used for several decades in agriculture, but also in housing for pest control^6^. Despite been globally discontinued in 1997, due to their ability to accumulate in the environment and to travel large distances from where they are released, chlordane-related compounds are present in soil, air, and water^7–9^. Although exposure levels of chlordane compounds are expected to decrease over time due to the decrease in their use, their harmful consequences may still be felt for very long periods. Moreover, a better understanding of their consequences may be essential to estimate the effect of other endocrine disruptors for which the effects were not know because exposure levels or time of exposure are still not sufficient to quantify their effects.

Chlordane compounds are endocrine disrupting chemicals (EDCs), which means that they may affect the natural function of hormones by blocking, mimicking, displacing, or acting to subvert their roles. There is increasing scientific evidence showing EDCs as risk factors in the pathogenesis and development of obesity and obesity-related metabolic syndrome such type 2 diabetes^10,11^. But there is no systematized information about the effect of chlordanes in humans. Since both obesity and diabetes are pathologies which occur progressively and do not have an immediate consequence after exposure, in addition to understanding the effect on disease occurrence, it is also important to understand the association with different levels of adiposity and diabetes-related features such as fasting glucose or insulin resistance. Previous studies preformed in animal models hypothesize that exposure to elevated levels of chlorane compounds increases the risk of adiposity and diabetes. As chlordane compounds are known as endocrine disruptores, it is possible that the exposition to these compounds, could also increase the risk of adiposity and diabetes in humans. However, to our knowledge, no systematic review has been performed regarding the association between the exposure to CHLs and adiposity or diabetes and diabetes-related features in humans.

We hypothesize that exposure to chlordane compounds increases the risk of adiposity and diabetes in humans. Thus, we used a PECO framework (population, exposure, comparator, and outcomes) to develop our question. PECO was applied as follows: P, general adult population; E, exposure to chlordane compounds; C, individuals without adiposity and diabetes; O adiposity and diabetes. These PECO statements were used to develop the search terms and inclusion/exclusion criteria for our systematic review in the next step to summarize the epidemiological data on the association between chlordane compounds and adiposity and diabetes using a systematic approach to identify and perform a meta-analysis of available data in order to evaluate our hypothesis.

## Methods

In 2016, our group started this work as part of the HEALS (Health and Environment-wide Associations based on Large population Surveys) project, producing a report that presents the preliminary results of the association between EDCs (which includes other EDC exposure) and adverse health outcomes related to adiposity and diabetes. The current publication is an update of the preliminary work, focusing specifically on the exposure to chlordane, using the methodology previously described^12^. This systematic review and meta-analysis was undertaken with a prospective protocol using the PRISMA (Preferred Reporting Items for Systematic Reviews and Meta-analyses) guidelines^13^ but was not registered in the PROSPERO database.

### Literature search

Pubmed and Web of Science were searched without imposing any limitations, from inception up to 15 February 2021, to retrieve original papers reporting data on the association between chlordanes, adiposity and diabetes, using the following search expressions:

Pubmed: (("Endocrine disruptor" OR Endocrine disruptor [mh] OR Pesticide OR "Brominated flame retardants" OR organochlorine OR chlordane* OR oxychlordane OR trans-nonachlor OR "heptachlor epoxide" OR trans-nonachlordane OR cis-chlordane OR cis-nonachlor) AND (Diabetes OR Glucose OR Glucose Metabolism Disorders [mh] OR glycemia OR hyperglycemia OR Hypoglycemia OR Hyperinsulinism OR "Insulin resistance" OR obesity OR overweight OR BMI OR "Body fat" OR Adipose tissue [mh] OR Body size [mh] OR "body size" OR "body weight" OR Anthropometry OR "anthropometric measures”)) AND (humans [mh]).

**Figure 1:**
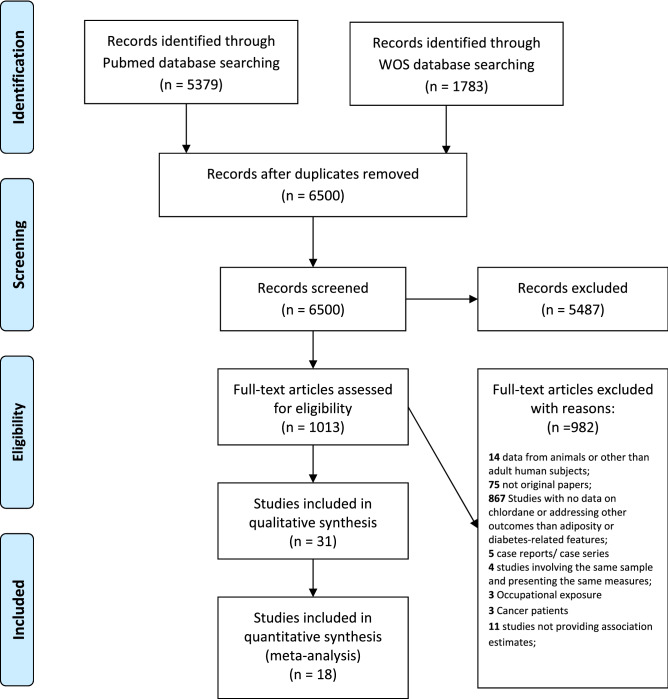
Systematic review flowchart.

ISI-Web of Science: *((TS* = *”Endocrine disruptor" OR TS* = *Pesticide OR TS* = *"Brominated flame retardants" OR TS* = *organochlorine OR TS* = *chlordane OR TS* = *oxychlordane OR TS* = *trans-nonachlor OR TS* = *"heptachlor epoxide" OR TS* = *trans-nonachlordane OR TS* = *cis-chlordane OR TS* = *cis-nonachlor) AND (TS* = *Diabetes OR TS* = *Glucose OR TS* = *“Glucose Metabolism Disorders” OR TS* = *glycemia OR TS* = *hyperglycemia OR TS* = *Hypoglycemia OR TS* = *Hyperinsulinism OR TS* = *"Insulin resistance" OR TS* = *obesity OR TS* = *overweight OR TS* = *BMI OR TS* = *"Body fat" OR TS* = *“Adipose tissue” OR TS* = *"body size" OR TS* = *"body weight" OR TS* = *Anthropometry OR TS* = *"anthropometric measures”)) AND (TS* = *humans)*. The systematic review flowchart is presented in Fig. 1.

### Article selection

References were reviewed independently by two of three researchers (CR, ID and VM) according to the following exclusion criteria: (1) studies not written in English, Portuguese or Spanish, (2) studies not involving adult human subjects, (3) studies not presenting original data (review articles, editorials, reports, comments or guidelines), (4) case reports or case series, (5) studies addressing exposures other than chlordanes or outcomes other than adiposity or diabetes-related features and (6) studies not providing association estimates, (7) studies performed in individuals with a diagnosed disease (e.g., tumors) and (8) samples composed exclusively by individuals with high exposure to chlordanes (e.g., workers involved in mixing or applying pesticides), since these individuals have presumably been exposed to much higher doses of chlordanes than individuals from other study samples, and therefore, exposure levels may not present enough variability to reflect the real association. When more than one study referred to the same sample, we considered the one presenting data for the largest sample or, if the sample size was equal, we chose the one providing results in more detail. On the other hand, if two publications studying the same sample reported complementary results (i.e. presenting estimates for different outcome measures), that otherwise would be lost if only one was included, both of them were considered eligible for data extraction^12^.

In step one, the exclusion criteria were applied to title and/or abstract and in step two, to the full-text. When the abstract of a particular paper was not available, the article was selected for evaluation in step two, except when the title unequivocally presented information for exclusion (non adult human subjects, review articles, editorials, reports, comments, guidelines or case reports). Decisions taken independently by the two reviewers were compared in both steps and disagreements were resolved by consensus or after discussion involving a fourth researcher (ER). The agreement between the reviewers was 68.1% in step one and 72.3% in step two^12^. The literature search was then complemented by backward citation tracking of the papers considered for data extraction.

Our search identified 6500 publications of which 31 presented original data on the association between chlordanes and adiposity or diabetes-related features.

### Data extraction

Three researchers (CR, ID and VM) have independently extracted information from selected studies: (1) study design (type of study and period of data collection), (2) sample characteristics (country, type of population, sex, age and sample size), (3) exposure characteristics (chlordane compound, biological source and assessment method), (4) outcome characteristics (adiposity measure, diabetes-related features and assessment method), (5) descriptive measures and (6) association measures. Estimates on the association between exposure to chlordanes and adiposity or diabetes-related features were extracted for the whole sample, but when available, age- or sex-stratified estimates were also retrieved^12^. Of the 31 studies evaluated, 18 were considered only for the meta-analysis (Fig. 1).

### Meta-analysis procedure

For the 31 papers included in the systematic review, additional exclusion criteria were considered for the meta-analysis in order to improve comparability between studies. Then, we excluded studies performed among pregnant women since pregnancy induces changes in adiposity and/or diabetes measures and may consequently modify the association and studies presenting association measures that were not possible to combine [e.g. odds ratios (ORs) with correlation coefficients]. Regarding the meta-analysis for adiposity, since most studies provided correlation or beta coefficients to estimate the association with chlordanes, we excluded studies presenting incompatible measures, such as ORs, relative risks or hazard ratios. We also excluded studies that provided coefficients for log-transformed variables since they were not comparable with those provided for untransformed variables, which were the most commonly described. Conversely, as the majority of papers on the association between chlordanes and diabetes-related features presented results using ORs, studies that provided beta or correlation coefficients were excluded from the meta-analyses. When the exposure was analyzed as a categorical variable, only the estimate concerning the highest exposure category in comparison with the lowest was included^12^.

Meta-analyses were performed whenever three or more eligible estimates evaluating the effect on adiposity measures and diabetes-related features were suitable to be combined. Therefore, meta-analyses were performed for the association of oxychlordane and trans-nonachlor with both adiposity and diabetes and for heptachlor epoxide exclusively with diabetes.

### Statistical analysis

For meta-analyses on the association between chlordanes and adiposity, standardized regression coefficients and its standard error were calculated in different research approaches of the original studies^14^. When necessary, regression coefficients and Pearson or Spearman correlation coefficients were converted and used for meta-analyses. We used the standard errors to compute the weight of each individual study. When comparing the standard errors obtained from beta coefficients to those calculated for correlation coefficients, minor differences were found (0.02 units on average). This contributes to the robustness of the results reported by our systematic review and meta-analysis. Appropriately converted data from studies were combined using fixed effects meta-analyses to compute summary regression coefficients and corresponding 95% confidence intervals (95% CI)^15^. In order to compute summary estimates (ORs) and respective 95% CIs for the available data on the association between chlordanes and diabetes-related features, the DerSimonian and Laird computational method was used. Heterogeneity between studies was assessed by the *I*^2^ statistic^16^. Visual inspection of the funnel plots and the Egger’s regression asymmetry test were used for assessment of publication bias^17^. We also performed sensitivity analysis in order to explore remaining confounding.The statistical analysis was performed with STATA®, version 11.2 (StataCorp, College Station, TX, USA)^12^.

### Quality assessment

For the assessment of methodological quality of the studies included, we used the “Strengthening the Reporting of Observational Studies in Epidemiology (STROBE) Statement: Guidelines for Reporting Observational Studies”. The STROBE statement presents a checklist of items that tought to be addressed when observational studies are reported. The STROBE Statement was used to evaluate the quality of reporting of included observational studies and their scores were assessed (Supplementary Table S1)^18,19^.

## Results

In this systematic review we have identified thirty one publications reporting on the association between chlordanes and adiposity and/or diabetes-related features, described in supplementary Table S2^20–50^. Twenty one papers assessed oxychlordane as the exposure^20–23,25–28,32–34,36–43,50^—ten on adiposity^20–23,25,26,37,39–41^ and thirteen on diabetes-related features^25–28,32–34,36,38,42,43,50^; twenty eight have focused on trans-nonachlor^20–34,36–43,45–47,49,50^—fifteen on adiposity^20–26,29,31,37,39–41,45,47^ and fifteen on diabetes^25–28,30,32–34,36,38,42,43,46,49,50^; five publications measured the association between heptachlor epoxide and diabetes-related features^34,35,48,50^. Starling, AP et al*.*^48^ was the only study assessing the association between chlordane and diabetes-related features. Cordier et.al^44^. was the only study providing associations between the sum of chlordanes and diabetes-related features (Supplementary Table S4). Adiposity was assessed by five different measures (Body Mass Index (BMI), fat mass, waist circumference, visceral adipose tissue and subcutaneous adipose tissue), with BMI being the most common measure across all compounds. The diabetes-related features were evaluated through self-reported diagnosis of diabetes, anti-diabetic medication use, medical records, the assessment of fasting and non-fasting blodd glucose, oral glucose tolerance test, HbA1c levels and insulin resistance (HOMA-IR).

### Adiposity

#### Oxychlordane and adiposity

**Figure 2:**
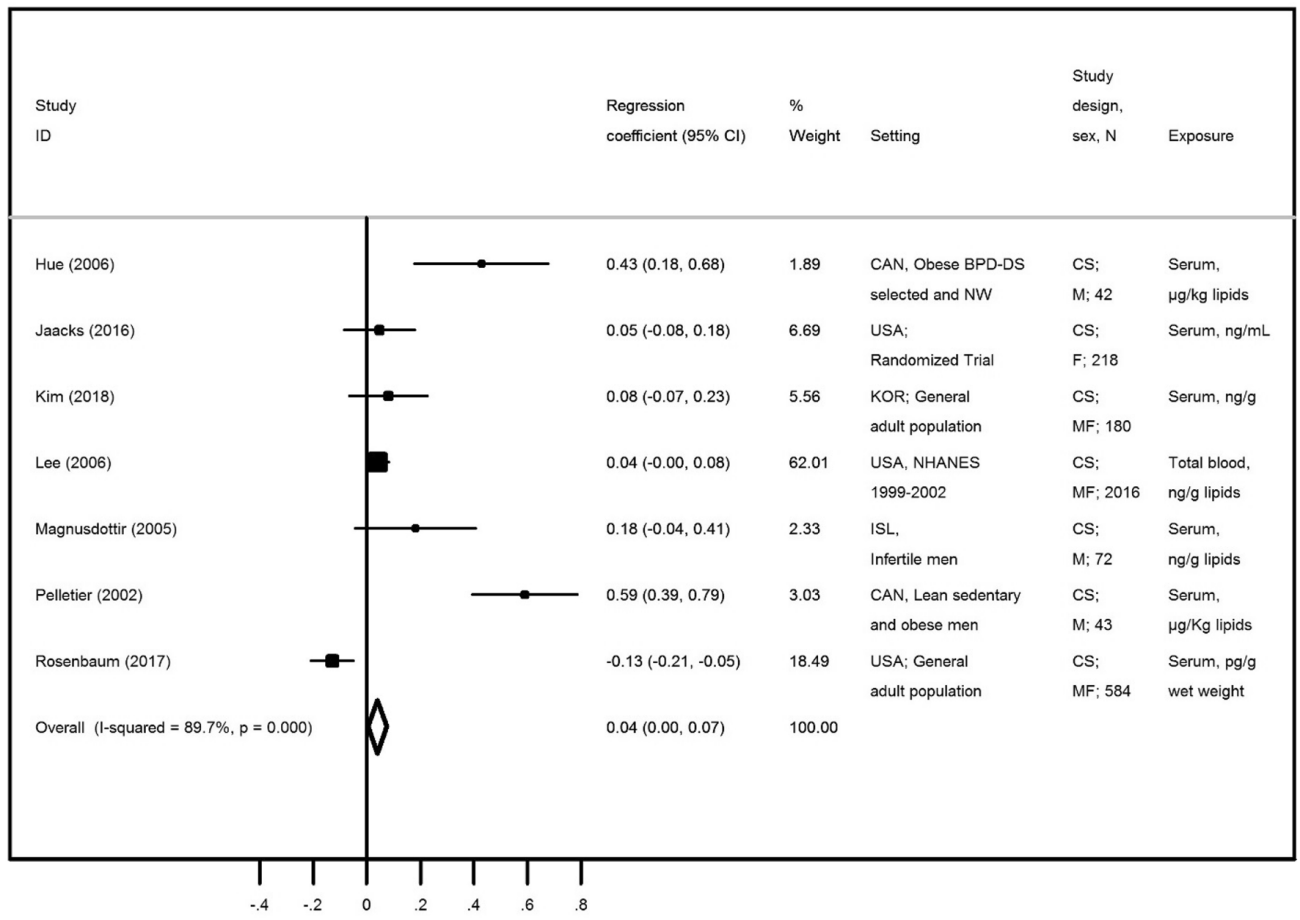
Meta-analysis of studies evaluating the association of oxychlordane with body mass index (BMI). Beta coefficients and corresponding 95% CI (horizontal lines). The size of the black square indicates the study’s weight in the analysis (weights are from fixed-effects analysis). The centre of the open diamond indicates the summary estimate and its width represents the 95% CI.

Table 1 presents data regarding the associations between oxychlordane and all the identified adiposity measures. Although we have found results for 3 different adiposity measures, a meta-analysis was only possible to be performed for BMI. Of the ten papers identified in the systematic review, three (Lee et al.^25^, Lee et al.^26^ and Cho et al.^23^) were performed using NHANES 1999–2000 and 2001–2002 combined datasets. The paper by Lee et al.^25^ was included in the meta-analysis since Lee et al.^26^ and Cho et al*.*^23^ did not assess BMI as the adiposity measure. Bjerregaard-Olsen^40^ has evaluated the association among pregnant women using pre-pregnancy BMI as outcome, and was thus excluded from the meta-analysis. We have included a total of seven publications in the meta-analysis^20–22,25,37,39,41^, all of them presenting cross-sectional data on the association between oxychlordane and BMI, with a sample size ranging from 42 to 2016 individuals. The overall beta coefficient was 0.04 (95% CI 0.00; 0.07) (Table 1 and Fig. 2), indicating a weak and no significant association with BMI. Heterogeneity between studies was very high (*I*^2^ = 89.7%). However, with the exception of Rosenbaum et al.^39^, all included estimates have shown positive associations. Visual inspection of the funnel plot (Supplementary Figure S1A) suggested an underrepresentation of small studies with negative associations and of large studies with positive associations, although the Egger’s regression asymmetry test (*p* = 0.193) showed no statistically significant publication bias.

**Table 1:** Description of all found associations between oxychlordane and each adiposity measure.

Publication characteristics			Adiposity		
			BMI (kg/m^2^)	Fat mass	WC (cm)
References	Sex, age group subjects	Exposure contrast #	Estimate (95% CI)	Estimate (95% CI)	Estimate (95% CI)
Pelletier et al.^20^	♂ (36–58)	μg/Kg lipids	*ρ = 0.53 (p < 0.001)*	ρ = 0.41 (*p* = 0.006)	
Magnusdottir et al.^21^	♂ (37 ± 5.4)*	ng/g lipid	*ρ = 0.18* *(p = 0.14)*		
Hue et al.^22^	♂ †	μg/kg lipids	*r = 0.42* *(p = 0.05)*		
Lee et al.^25^	♂♀ (≥ 20)	ng/g lipid ≥ 90th vs. BDL	*ρ = 0.04* *(p > 0.01)*		ρ = 0.01 (*p* > 0.01)
Lee et al.^27^	♂♀ (≥ 20)	> 75th vs. BDL			OR = 1.2 (0.6; 2.4)
Cho et al.^23^	♂ (< 50)	ng/g lipids		r = 0.01 (*p* > 0.05)	
Cho et al.^23^	♂ (≥ 50)	ng/g lipids		r = 0.19 (*p* < 0.001)	
Cho et al.^23^	♀ (< 50)	ng/g lipids		r = − 0.11 (*p* < 0.01)	
Cho et al.^23^	♀ (≥ 50)	ng/g lipids		r = 0.01 (*p* > 0.05)	
Jaacks et al.^41^	♀ (18–40)	ng/mL	*ρ = 0.05* *(p = 0.43)*		
Bjerregaard-Olesen et al.^40^	♀ (26–32)	μg/kg lipids	**#** β = 0.00 (− 0.03; 0.02)		
Rosenbaum et al.^39^	♂♀ (53.6 ± 16.2)	pg/g wet weight	*ρ = − 0.134*		
Kim et al.^37^	♂♀ (41–69)	ng/g lipid	*r = 0.08* *(p > 0.05)*		
Meta-analysis			**β = 0.04** **(0.00; 0.07)**		

BMI, Body Mass Index; WC, Waist Circumference; β, Beta coefficient; OR, Odds Ratio; ρ, spearmen correlation, r, Pearson correlation; * Mean; †, Without information; # Pre pregnancy-BMI as outcome; italic values represents studies for which was possible to perform a meta-analysis plot.

We have also performed a sensitivity analysis by stratifying data according to the year of publication. Considering studies published before 2006, a positive and statistically significant association between oxychlordane and BMI [β = 0.08 (95% CI 0.04; 0.12); *I*^2^ = 91.9%] was found, while more recently published studies did not find an association between oxychlordane and BMI [β = − 0.05 (95% CI − 0.11; 0.01); *I*^2^ = 75.5%)].

The estimates for other adiposity measures were consistent with the associations described for BMI (Table 1 and Supplementary Table S3). Regarding fat mass, only two papers presented results but Cho et al.^23^ provided stratified results by age and sex, which allows to have five estimates for the association between oxychlordane and fat mass. The overall beta coefficient for the five estimates provided by the two papers evaluating fat mass^20,23^ was indeed similar to that found for BMI [β = 0.03 (95% CI − 0.01; 0.07); *I*^2^ = 90.0%). The two papers presenting data for the association between oxychlordane and waist circumference^25,26^ were based on the same population (NHANES study 1999–2002). The result from the less restrictive analysis showed a positive and significant association, as found for the same study regarding BMI.

#### Trans-nonachlor and adiposity

**Figure 3:**
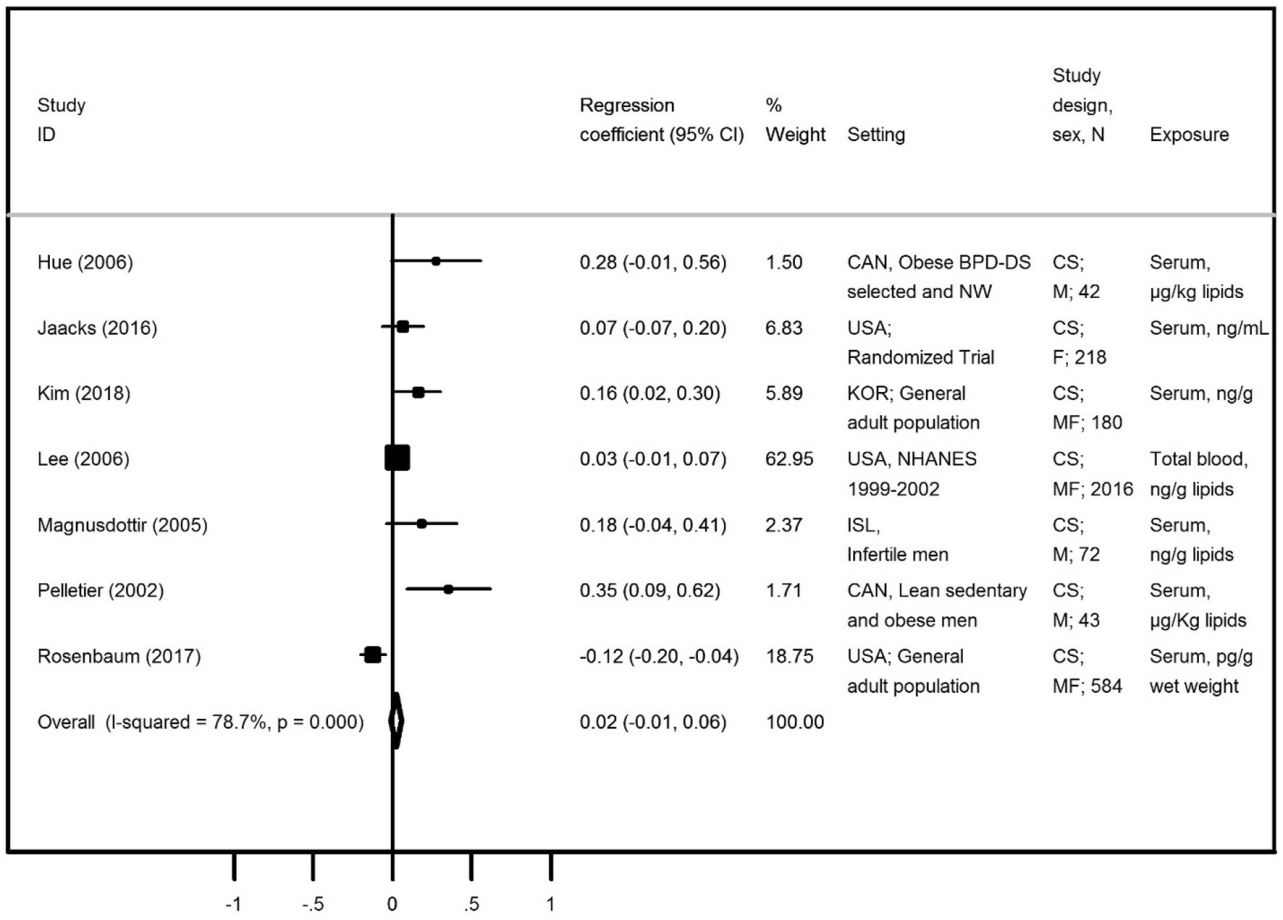
Meta-analysis of studies evaluating the association of trans-nonachlor with body mass index in adults. Beta coefficients and corresponding 95% CI (horizontal lines). The size of the black square indicates the study’s weight in the analysis (weights are from fixed-effects analysis). The centre of the open diamond indicates the summary estimate and its width represents the 95% CI.

The description of papers assessing the association between trans-nonachlor and adiposity measures is presented in Table 2. A total of nine papers presented data focusing on BMI and were included seven in the meta-analysis^20–22,25,37,39,41^, all using cross-sectional data. We found a high heterogeneity level (78.7%) and the visual inspection of the funnel plot (Supplementary Figure S2A) suggested an underrepresentation of small studies, although the Egger’s regression asymmetry test (*p* = 0.183) supported no statistically significant publication bias. The majority of results presented a positive association between trans-nonachlor and BMI but the overall beta coefficient was 0.02 (95% CI − 0.01; 0.06) (Fig. 3). Rosenbaum et al.^39^ reported negative and statistically significant associations β = − 0.12 (95% CI − 0.20; − 0.04), and accounted for 18.75% of total weight for the overall coefficient.

**Table 2:** Description of all found associations between trans-nonachlor and adiposity measures.

Publication characteristics			Adiposity			
			BMI (kg/m^2^)	Fat mass	WC (cm)	VAT/SAT
References	Sex. Age group Subjects	Exposure Contrast #	Estimate (95% CI)	Estimate (95% CI)	Estimate (95% CI)	Estimate (95% CI)
Pelletier et al.^20^	♂ (36–58)	μg/Kg lipids	*ρ = 0.34 (p = 0.02)*	ρ = 0.23 (*p* = 0.143)		
Magnusdottiret al.^21^	♂ (37 ± 5.4)*	ng/g lipid	*ρ = 0.18 (p = 0.12)*			
Hue et al.^22^	♂ †	μg/kg lipids	*r = 0.28 (p = 0.07)*			
Lee et al.^25^	♂♀ (≥ 20)	ng/g lipid ≥ 90th vs. BDL	*ρ = 0.03 (p > 0.01)*		ρ = 0.01 (*p* > 0.01)	
Lee et al.^27^	♂♀ (≥ 20)	> 75th vs. BDL			OR = 1.4 (0.7; 2.9)	
Cho et al.^23^	♂ (< 50)	ng/g lipids		r = 0.01 (*p* > 0.05)		
Cho et al.^23^	♂ (≥ 50)	ng/g lipids		r = 0.11 (*p* < 0.01)		
Cho et al.^23^	♀ (< 50)	ng/g lipids		r = − 0.13 (*p* < 0.001)		
Cho et al.^23^	♀ (≥ 50)	ng/g lipids		r = 0.04 (*p* > 0.05)		
Ronn et al.^24^	♂♀ (70y.o.)	Per 1-unit (ln ng/g lipid)		β = 1.86 (0.85; 2.86)		
Lee et al.^29^	♂ (70y.o.)	370–4252 vs. < 173 (pg/ml)			OR = 2.5 (1.1; 5.6)	
Lee et al.^29^	♀ (70y.o.)	370–4252 vs. < 173 (pg/ml)			OR = 0.9 (0.5; 1.8)	
Lee et al.^29^	♂ (≥ 75)	370–4252 vs. < 173 (pg/ml)			OR = 0.7 (0.2; 2.4)	
Lee et al.^29^	♀ (≥ 75)	370–4252 vs. < 173 (pg/ml)			OR = 0.7 (0.2; 1.9)	
Roos et al.^31^	♂♀ (70y.o.)	Per 1-unit (ln ng/g lipid)				VAT: β = 17.0 (4.7; 30.0) SAT: β = 19.0 (− 2.6; 40.0)
Jaacks et al.^41^	♀ (18–40)	ng/mL	*ρ = 0.07 (p = 0.27)*			
Lauritzen et al.^47^	♀ (18–41) Pregnant	Per 1-unit (ln ng/g lipid)	**#** β = − 2.0 (− .4; − 0.6)			
Bjerregaard-Olesen et al.^40^	♀ (26–32) Pregnant	μg/kg lipids	**#** β = − 0.02 (− 0.04; 0.00)			
Rosenbaum et al.^39^	♂♀ (53.6 ± 16.2)	pg/g wet weight	*ρ = − 0.129*			
Kim et al.^37^	♂♀ (41–69)	ng/g lipid	*r = 0.16* *(p < 0.05)*			
Chen et al.^45^	♀ (19–40) Pregnant	ng/g lipid	**#** OR = 1.19 (0.42; 3.33)			
Meta-analysis			**β = 0.02 (− 0.01; 0.06)**	–	**–**	**–**

BMI, Body Mass Index; WC, Waist Circumference;VAT, Visceral adipose tissue, SAT Subcutaneous adipose tissue; β, Beta coefficient; OR, Odds Ratio; ρ, spearmen correlation; r, Pearson correlation; * Mean; †, Without information; # Pre pregnancy-BMI as outcome; italic values represents studies for which was possible to perform a meta-analysis plot.

Besides BMI, data on fat mass, waist circumference, visceral and subcutaneous adipose tissue are presented also in Table 2. Pelletier et al.^20^, Cho et al.^23^ and Ronn et al.^24^ provided six estimates evaluating the association regarding fat mass. In general, all results were positive but not statistically significant. The summary measure of these associations, excluding Ronn et al.^24^ for using *ln*-transformed variables, was 0.01 (95% CI − 0.03; 0.04), with an heterogeneity of 81.7% (Supplementary Table S3). Regarding waist circumference, all data were obtained from two samples (NHANES and PIVUS) and mixed results were observed—positive, but not significant, associations were found in NHANES^25,26^ while Lee et al.^29^ found mostly negative associations among PIVUS participants. Positive associations were observed in the single study that assessed visceral and subcutaneous adipose tissue^31^, but with great uncertainty expressed by the very wide 95% CI (Table 2).

### Diabetes-related features

Diabetes-related features were evaluated by the self-reported diagnosis of diabetes, anti-diabetic medication use, medical records, the assessment of fasting and non-fasting blodd glucose, oral glucose tolerance test, HbA1c levels and insulin resistance (HOMA-IR). The meta-analysis perfomed in this topic includes estimates for all diabetes-related features.

#### Oxychlordane and diabetes-related features

**Figure 4:**
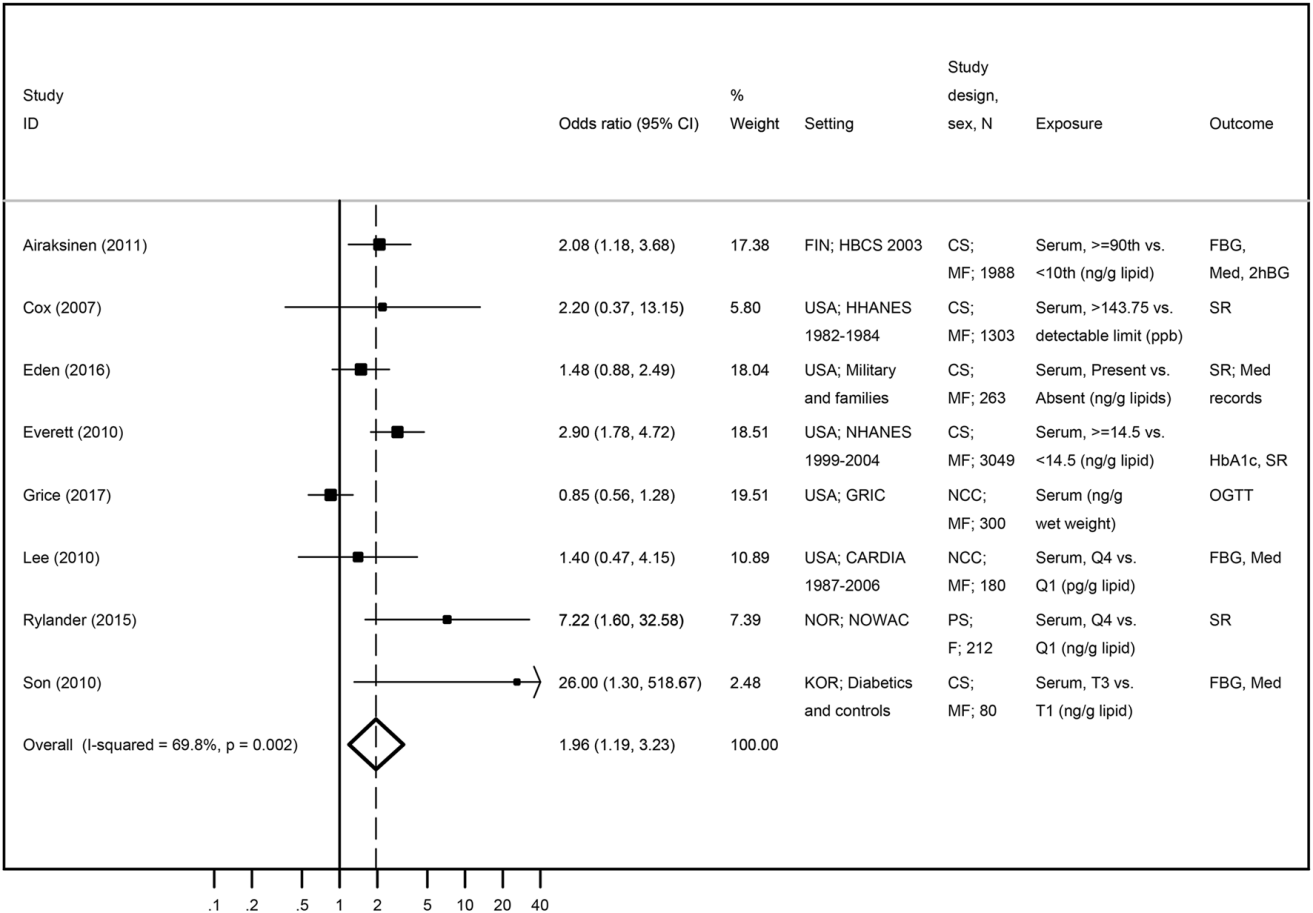
Meta-analysis of studies evaluating the association of oxychlordane with diabetes-related features. Odds ratios and corresponding 95% CI (horizontal lines). The size of the black square indicates the study’s weight in the analysis (weights are from fixed-effects analysis). The centre of the open diamond indicates the summary estimate and its width represents the 95% CI.

The description of studies selected for the systematic review on the association between oxychlordane and diabetes-related features is found in Table 3. From the thirteen papers identified in the systematic review, four reported data on NHANES 1999–2002^25–27,34^ and Zong et al.^43^ included women from NHANES 1999–2006. In order to avoid the overrepresentation of this population, only Everett and Matheson^34^ was included in the meta-analysis for being the one with the largest sample size. Everett and Matheson^34^ presented data for two outcome categories (diabetes and pre-diabetes), but only the estimate for diabetes was included in the meta-analysis. Park et al.^50^ was also excluded for presenting correlation coefficients as the association measure instead of OR, the most used estimate in the other papers. Eight studies were included in this meta-analysis^28,32–34,36,38,42^. Five presented cross-sectional data, while three followed a longitudinal approach^28,36,38^. With the exception of Grice et al.^38^, all results showed a positive association with diabetes, with an overall OR of 1.96 (95% CI 1.19; 3.23, *I*^2^ = 69.8%) (Fig. 4). All estimates identified by the systematic review but excluded in the meta-analysis suggested a positive association, which also supports the results drawn from the meta-analysis. As Son et al.^49^ and Rylander et al*.*^36^ presented unusually high estimates, we conducted a sensitivity analysis excluding both studies. As the association was stronger [OR = 2.37 (95% CI 1.68; 3.34), *I*^2^ = 0.0%], we decided to maintain a conservative approach and keep both studies in the analysis. Visual inspection of the funnel plot (Supplementary Figure S1B) suggested an underrepresentation of small studies, although the Egger’s regression asymmetry test (*p* = 0.164) showed no statistically significant publication bias.

**Table 3:** Description of all found associations between chlordane compounds and diabetes-related features.

Publication characteristics				Chlordane compound			
				Oxychlordane	Trans-nonachlor	Heptachlor	Chlordane
References	Sex. Age group subjects	Diabetes	Exposure Contrast #	Estimate (95% CI)	Estimate (95% CI)	Estimate (95% CI)	Estimate (95% CI)
Lee et al.^25^	♂♀ (≥ 20)	FBG or NFBG or Self-reported	≥ 90th vs. BDL	**OR = 6.50** **(1.99; 21.26)**	**OR = 11.8** **(4.4; 31.3)**		
Cox et al.^33^	♂♀ (20–74)	Self-reported	> 143.75 vs- detectable limit (ppb)	*OR = 2.20* *(0.37; 13.15)*	*OR = 1.50* *(0.43; 5.25)*		
Lee et al.^26^	♂♀ (20–85)	HOMA-IR	> 75th vs. BDL	**OR = 8.70** **(2.29; 33.10)**	**OR = 5.40** **(1.28; 22.76)**		
Lee et al.^27^	♂♀ (≥ 20)	FBG	> 75th vs. BDL	OR = 3.1 (0.80; 11.90)	OR = 7.00 (0.81; 60.42)		
Everett et al.^34^	♂♀ (≥ 20)	HbA1c	≥ 14.5 vs. < 14.5 (ng/g lipid)	*OR = 1.28* *(0.88; 1.87)*	*OR = 1.30* *(0.88; 1.9)*	*OR = 1.45* *(1.04; 2.01)*	
Lee et al.^28^	(20–36)	FGB. Med	Q4 vs Q1 (pg/g lipid)	*OR = 1.40* *(0.47; 4.15)*	*OR = 2.00* *(0.59; 6.78)*		
Park et al.^50^	♂♀ w ms (56.5 ± 6.9)*	HOMA-IR	log ng/g lipid	r = 0.08 (*p* > 0.05)	r = 0.06 (*p* > 0.05)	r = 0.20 (*p* > 0.05)	
Park et al.^50^	♂♀ w/o ms (56.5 ± 6.9)*	HOMA-IR	log ng/g lipid	r = 0.25 (*p* > 0.05)	r = 0.20 (*p* > 0.05)	**r = 0.32** **(*p* < 0.05)**	
Patel et al.^35^	♂♀ †	FBG	Per SD (log ng/g)			**OR = 1.70** **(1.30; 2.10)**	
Lee et al.^30^	♂♀ (≥ 70)	FBG. Med	FBG ≥ 112 mg/dL or medication		*OR = 1.80* *(0.49; 6.64)*		
Son. et al.^49^	♂♀ †	FBG. Med	T3 vs. T1 (ng/g lipids)	***OR = 26.00*** ***(1.30; 518.87)***	***OR = 8.10*** ***(1.21; 54.08)***	*OR = 3.10* *(0.80; 12.06)*	
Airaksinen et al.^32^	♂♀ (62)*	FBG. Med. OGTT	≥ 90th (19.0–76.0) vs. < 10th (0.73–6.3) (ng/g lipids)	***OR = 2.08*** ***(1.18; 3.86)***	***OR = 2.24*** ***(1.25; 4.02)***		
Starling et al.^48^	♀ (17–88)	Self-reported	Exposed vs. Non-exposed			*OR = 1.48* (*0.92;* *2.37*)	**OR = 0.95** **(1.01; 1.34**)
Rylander et al.^36^	♀ (30–70)	Self-reported	Q4 vs. Q1 (ng/g lipids)	***OR = 7.22*** ***(1.6; 32.58)***	***OR = 6.56*** ***(1.57; 27.45)***		
Eden et al.^42^	♂♀ (18–65)	Self-reported. Medical records	Present vs. Absent (ng/g lipids)	*OR = 1.48 (0.88; 2.49)*	***OR = 2.25 (1.18; 4.29)***		
Zong et al.^43^	♀ †	Self-reported. Med	T3 vs T1	OR = 2.42 (0.51; 9.86)	**OR = 4.44** **(1.08; 18.20)**		
Grice et al.^38^	♂♀ †	OGTT	(ng/g wet weight)	*OR = 0.85* *(0.56; 1.27)*	*OR = 1.06* *(0.68; 1.64)*		
Han et al.^46^	♂♀	FBG. Med	T3 vs T1 (ng/g lipids)		***OR = 6.29 (3.20; 12.36)***		
Meta-analysis				**OR = 1.96** **(1.19; 3.23)**	**OR = 2.43** **(1.64. 3.62)**	**OR = 1.88** **(1.42; 2.49)**	–

β, Beta coefficient; OR, Odds Ratio; r, Pearson correlation; * Mean; †, Without information; w/ ms, whith metabolic syndrome; w/o ms, without metabolic syndrome; FBG, fasting blood glucose; NFBG, nonfasting blood glucose; HOMA-IR, Homeostatic model assessment—insulin resistance; Hb1A1c, hemoglobin A1c; OGTT, glucose tolerance test; Med, Medication; italic values represents studies included in meta-analysis.

#### Trans-nonachlor and diabetes-related features

**Figure 5:**
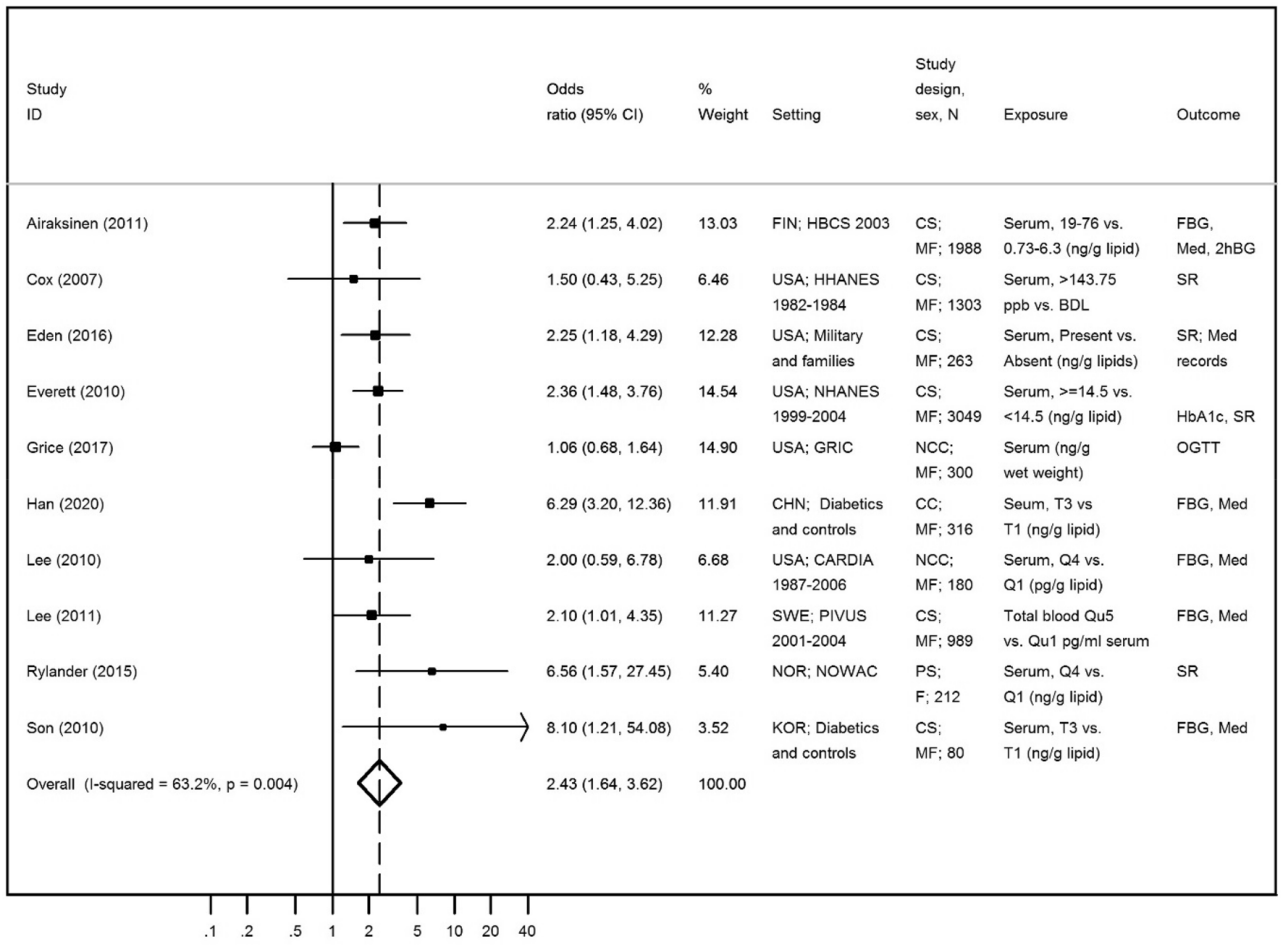
Meta-analysis of studies evaluating the association of trans-nonachlor with diabetes-related features in adults. Odds ratios and corresponding 95% CI (horizontal lines). The size of the black square indicates the study’s weight in the analysis (weights are from fixed-effects analysis). The centre of the open diamond indicates the summary estimate and its width represents the 95% CI.

The fifteen studies identified in the systematic review assessing the association between trans-nonachlor and diabetes are described in Table 3. Five of them reported data from the NHANES study^25–27,34,43^ . Similarly, in order to avoid the overrepresentation of this population, only Everett, CJ and Matheson^34^ was included in the meta-analysis for being the one with the largest sample size. This study presented data for two outcome categories (diabetes and pre-diabetes), but only the estimate for diabetes was included. Park et al.^50^ was not selected for inclusion in the meta-analysis since estimates were presented only as correlation coefficients, thus ineligible. Lee et al*.*^23^ presented both cross-sectional and prospective results, but only cross-sectional estimates were considered for analysis since it was the most comparable estimate with the ones provided in other studies. Ten studies were then considered for the meta-analysis^28,30,32–34,36,38,42,46^, resulting in a positive and statistically significant overall estimate [OR = 2.43 (95% CI 1.64; 3.62), *I*^2^ = 63.2%) (Fig. 5). A sensitivity analysis was performed to test the exclusion of Son et al.^49^ and Rylander et al.^36^, since they presented a remarkably stronger association with low precision. However, as the exclusion resulted in a stronger association (OR = 2.19, 95% CI 1.47; 3.25; *I*^2^ = 65.2%), we have decided to include both studies in the final analysis. Visual inspection of the funnel plot (Supplementary Figure S2B) suggested an underrepresentation of small studies with negative associations, although the Egger’s regression asymmetry test (*p* = 0.178) showed no statistically publication significant bias. Regarding excluded estimates from the meta-analysis, no impact is expected since, with the exception of Park et al.^50^, all results were obtained from population samples already represented in the overall estimate. In adddition, all estimates not included in the meta-analysis also presented a positive association between trans-nonachlor and diabetes-related features, supporting the result observed.

#### Heptachlor Epoxide and diabetes-related features

**Figure 6:**
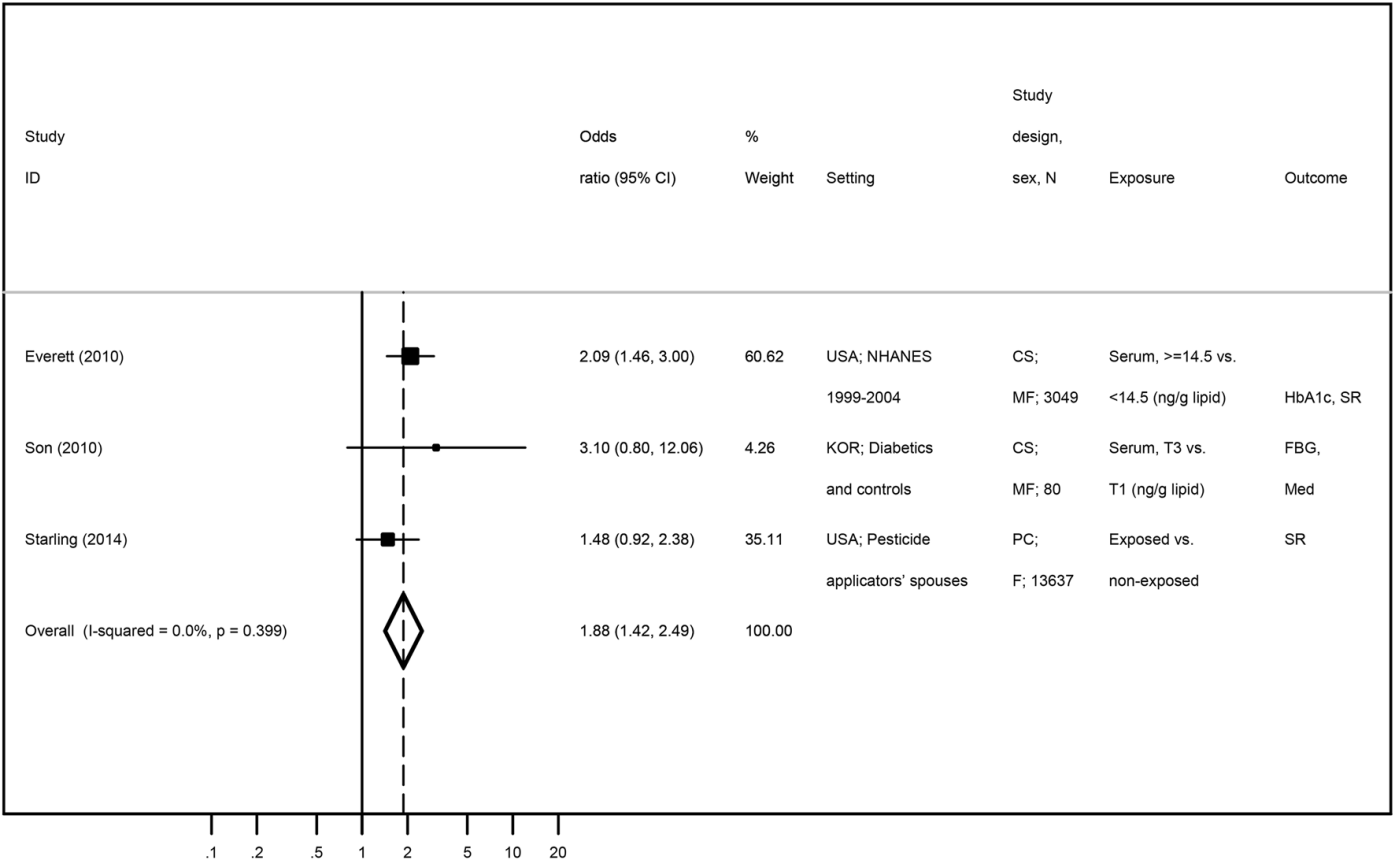
Meta-analysis of studies evaluating the association of heptachlor epoxide with diabetes-related features in adults. Odds ratios and corresponding 95% CI (horizontal lines). The size of the black square indicates the study’s weight in the analysis (weights are from fixed-effects analysis). The centre of the open diamond indicates the summary estimate and its width represents the 95% CI.

Five publications evaluated the association between heptachlor epoxide and diabetes-related features (Table 3)^34,35,50^. Two publications were performed among NHANES 1999–2004 participants^34,35^ and only Everett and Matheson^34^ was included in the meta-analysis for being the one with the largest sample size. This paper provided data for two outcome categories (diabetes and pre-diabetes), but only the estimate for diabetes was included. Park et al.^50^ was excluded for presenting association estimates as correlation coefficients instead of ORs. Three publications were then included in the meta-analysis^34,48,49^. The overall estimate on the association between heptachlor epoxide and diabetes was positive and statistical significant, although weaker than those found for oxychlordane and trans-nonachlor [OR = 1.88 (95% CI 1.42; 2.49)] (Fig. 6). However, the estimate found in Son et al.^49^ is high but with a low precision, while Starling et al.^48^ presented a lower risk estimate, probably because the classification of exposure was based on self-reported information and considerenig ever use of pesticides (life time exposure) which may occur long time before the study. As the number of included studies was very low, we were not able to perform sensitivitvy analyses, but despite the above mentioned study differences, heterogeneity was null (*I*^2^ = 0.0%). Visual inspection of the funnel plot (Supplementary Figure S3 suggested an underrepresentation of small studies, although the Egger’s regression asymmetry test (*p* = 0.806) showed no statistically significant publication bias.

#### Chlordane and diabetes-related features

Only one paper provided estimates on the exposure to technical chlordane and have evaluated its association with diabetes-related features (Table 3). The study by Starling et al.^48^ was performed among spouses of pesticide applicators and has shown an inverse association with diabetes (OR = 0.95, 95% CI 0.73; 1.23).

## Discussion

We have conducted a systematic review and meta-analysis presenting the most updated epidemiological evidence on the association between chlordanes, adiposity and diabetes-related features in adults. This systematic review was not registered a priori in the International Prospective Register of Systematic Reviews (PROSPERO). This is a limitation since it could introduce potential bias to the review. However our results were reported according PRISMA statement^13^ in order to minimize possible bias. The overall results suggest no association between chlordane compounds and adiposity but show higher odds of having diabetes-related features with increasing levels of all the chlordane compounds evaluated. Although these results may seem contradictory, it is necessary to emphasize that unlike results that we found in the case of diabetes, most studies assessing adiposity measures were not specifically designed to study the effect of chlordanes on adiposity, rather, they used adiposity measures as a covariate to test other hypothesis. In addition, most of the results studying the association between chlordanes and adiposity are based on continuous variables, using betas or correlation coefficients, while diabetes and its related features were mostly classified as categorical variables and the association quantified by ORs. Stronger associations were found in studies where the exposition variable is categorical rather than continuous, indicating that the observed differences in results between diabetes and adiposity may be related to the level of exposure. Even among studies addressing diabetes, the strength of association was stronger among those for which the exposure is analyzed as a categorical variable. The differences on identified studies estimating the association between chlordane compounds, adiposity and diabetes, are also reflected in the levels of heterogeneity observed across summary estimates, which are substancially higher for adiposity and therefore should be read with caution.

Since the identified studies used different measures of association to quantify the relationship between chlordanes and both adiposity and diabetes, it was not possible to include all the identified estimates in meta-analyses, which also impacts the robustness of our results. The small number of studies also limits the possibility to perform sensitivity analyses and to adequatly identify possible sources of variance between studies. Although we are not able to fully address the possible impact of these variables on our results, we can speculate on some of the potential sources of variance. Assay methodologies used for exposure assessment were quite diverse, which may lead to differences on the validity and precision of exposure assessments and increase heterogeneity between studies. The choice of different methods to measure adiposity and diabetes related features may also have implications for the overall estimates. Another aspect possibly conditioning the high heterogeneity observed is linked to different exposure levels between study samples, which is difficult to evaluate if the information is missing or presented in different formats. For instance, regarding adiposity, Lee et al*.*^25^ and Hue et al*.*^22^ did not present the mean values for oxychlordane nor for trans-nonachlor concentration levels, while Magnusdottir et al*.*^21^ and Pelletier et al*.*^20^ presented geometric and arithmetic means, respectively, and therefore we are not able to accurately evaluate the comparability between studies and the impact of different exposure levels on our results. Additionally, some confounders were recognized for the association between chlordanes and obesity and diabetes, being sex, age, race/ethnicity or dietary habits considering among the most important ones^51^. Although several publications presented adjusted estimates, which allow to assume that the observed associations are independent of these variables, some studies failed to take them into account.

In addition, it is also important to highlight that people with higher exposure levels to chlordane compounds may also be exposed to olther organochlorine substances, which makes it difficult to disentangle the specific effect of each chlordane compound. Since included studies did not presented the effect for each compound independently of other compounds, our meta-analyses will be not able to quantify the specific effect of each compound. Thus, it is not possible to ensure that at least some of the effect observed are not due exposure to EDCs other than chlordanes.

Despite these limitations, reliable conclusions can be drawn from the analyses summarizing the association between chlordane compounds and diabetes, which presented low heterogeneity levels and showed a consistent positive association through all the compounds evaluated. Although no statistical evidence of publication bias was observed, overall our results are suggestive of the existence of an underrepresentation of small studies.

### Adiposity

In order to gain a comprehensive perspective regarding the effect of chlordanes on adiposity, all the identified adiposity measures were considered eligible for analysis. We found estimates for BMI, fat mass, waist circumference, visceral and subcutaneous adipose tissue but meta-analyses were only possible using BMI. Nevertheless, neither oxychlordane nor trans-nonachlor showed a statistical significant positive association with BMI. As described above most studies assessing BMI were not specifically designed to study the effect of chlordanes on adiposity but presented this association as adiposity is a potential confounder for its specific aim. Also, for BMI, some studies used self-reported antropometric measures (height and weight). It is knonw that self-reported data are often not reliable, perhaps due to lack of recall or a desire to conform to aspired norms, which may increases heterogeneity and contribut to underestimated a possible association. In addition to the limitations described above, we would like to further emphasize two aspects. Firstly, chlordanes are lipophilic substances and may therefore accumulate in the adipose tissue. In cross-sectional studies whith exposure assessed using a blood sample, as in most of the studies identified by the systematic review, it is possible that individuals with higher percentage of body fat may present lower levels of exposure concentration, which will in turn decrease the magnitude of the association between the concentration of chlordanes and adiposity. Secondly, after stratifying our results according to the year of publication, we observed that older studies presented a positive and statistically significant association between oxychlordane and BMI, unlike more recent studies. This can be explained due to the progressive control of chlordane use over time. Chlordane started to be used in agriculture, then restricted for termite control and finally banned, based on concerns regarding its potential to cause cancer and its slow breakdown in the environment^52^. The lack of association evidenced in more recent studies may not mean that these compounds have no impact on obesity, but rather be a consequence of control measures regarding chlordane that have been implemented and that have neutralized the levels of chlordanes in the population.

### Diabetes-related features

The molecular mechanisms linking chlordanes and diabetes or diabetes-related features are currently poorly understood and only a few studies have addressed these effects. In vitro studies showed that members of the chlordane family may act via multiple signaling pathways, which icludes the activation of several nuclear receptors. Data based on laboratory studies investigating the potential mechanisms of action of chlordane effects on diabetes or diabetes-related features are presented in Supplementary data S1 and Supplementary Table S5.

In our study, we found a positive and statistically significant association for chlordane compounds and diabetes-related features. However, and particulalrly for oxychlordane and trans-nonachlor, moderate levels of heterogeneity were observed (69.8% and 63.2%, respectively). Possible reasons for these inconsistencies are explored below.

The outcomes used to assess diabetes included self-reported diagnosis of diabetes, anti-diabetic medication use, medical records, the assessment of fasting and non-fasting blood glucose, oral glucose tolerance test, HbA1c levels and insulin resistance (HOMA-IR) or the combination of more than one of these indicators. The lack of a consistent standardized approach to measure the outcome may contribute to the observed heterogeneity between studies. The use of ORs to estimate the association between chlordanes and diabetes has the disadvantage of requiring the comparison with a reference group, which is usually the one corresponding to the lowest category of exposure. By following this approach, estimates provided in different study populations may be difficult to compare since different populations may have different exposure levels and present a different risk in the reference category, even if the same measure of diabetes is being used. The impact of these differences on overall estimates was difficult to take into account since most studies did not provide descriptive data on exposure levels. In order to minimize the possible impact of this effect, we have excluded publications performed among individuals that were expected to have particularly high chlordane exposure levels, such as occupationally exposed populations. In addition, whenever a study presented estimates for categorized exposure levels, e.g. quantiles, we have chosen to include in the analysis the result for the highest category of exposure. This may be a relevant issue for our results since it has been suggested that for some EDCs, lower exposures may have stronger adverse effects on diabetes or diabetes-related features^53^. However, the current available information regarding chlordanes does not allow to understand if the association with diabetes is dependent on the exposure level. Another limitation of our results pertains to the fact that a significant number of included studies presented cross-sectional data, making it difficult to understand the temporal relationship between the exposure and the outcome. Type 2 diabetes may alter the metabolism of EDCs, either by slowing their excretion rate or by increasing the release of these substances from adipose tissue into the blood stream, by inducing lipolysis. In any case, the presence of glucose metabolism disorders may affect the measured levels of chemicals and not the other way around, as it would be intended to investigate in this study. However, this effect tends to decrease the strength of the association, and thus, the positive association observed in our results may be underestimated. In addtion, there were some inconsistencies observed regarding the adjustment for confounding effects across studies, particularly concerning oxychlordane and trans-nonachlor. Three of the included studies did not provide adjusted estimates for the association between these compounds and diabetes and although the majority has taken into account factors like age and BMI among their estimates, many failed to adjust for other potential variables of interest such as sex, race or lipidic profile. The adjustment for blood lipids is however a controversial issue, considering that on the one hand, chlordanes are lipophilic substances and higher lipid blood levels may lead to a higher chemical concentration, which would in turn lead to overestimated associations if this is not properly taken into account in regression models. On the other hand, it is also known that the exposure to these POPs may lead to higher lipid levels which are also associated with diabetes. This would mean that lipids could be a mediator on the association between EDCs and diabetes and thus lipid-adjusted models would not be recommended. However, and despite these limitations, the consistency found for a positive association between the exposure to chlordanes and diabetes across all the compounds identified allows us to be confident in our results.

### Future perspectives

One of the major literature gaps identified is the lack of longitudinal data evaluating the association between chlordanes and both adiposity and diabetes/diabetes-related features. Only two papers with a longitudinal approach have measured the association between chlordanes and adiposity (although both were performed among PIVUS participants)^24,29^, while three longitudinal studies have assessed diabetes^28,30,48^. As future perspectives, we propose that more studies need to be conducted following a longitudinal approach and including large, and representative samples. New evidence should also include the evaluation of dose–response relationships and the role of joint exposures. Exposure to chlordanes should be directly measured in appropriate biological samples at multiple time points throughout follow-up and according to standardized procedures. Information on key confounders should also be systematically collected and accounted for in future research.

In conclusion, this revision for the first time allow us to have data that support that odds of having diabetes significantly increase with increase levels of chlordanes but the data did not allow to reach a clear conclusion regarding association with adiposity in humans. For most of the analyzed compounds, the other studies identified in the systematic review also showed results in accordance with those found in meta-analyses. Although our overall estimates do not reveal a strong association between these compounds and adiposity or diabetes, considering the ubiquitous use of these chemicals, even if the increased risk is small, its impact on public health will still be relevant. The consistency of results across the assessed compounds gives us confidence in our meta-analyses results, although, in general, evidence is still scarce to draw firm conclusions. Another general comment is linked to the difficulty of establishing comparisons between studies due to unstandardized methodological choices. An international agreement on methods to measure both exposure and outcome variables and to conduct epidemiological studies could increase the knowledge on how adverse effects of exposure to various stressors (exposome) can influence human health.

## Supplementary Information

Supplementary Information.

## Data Availability

The datasets used and/or analysed during the current study are available from the corresponding author on reasonable request.
