# Supplementary material for: The association between environmental exposures to chlordanes, adiposity and diabetes-related features: a systematic review and meta-analysis
Source: Sci Rep. 2021 Jul 15;11:14546. doi: 10.1038/s41598-021-93868-4 (PMC8282629; doi:10.1038/s41598-021-93868-4)
Supplement: Supplementary file 1 — Supplementary Information. [file 41598_2021_93868_MOESM1_ESM.docx]

**The association between environmental exposures to chlordanes, adiposity and diabetes-related features: a systematic review and meta-analysis**

Vânia Mendes^1#^, Cláudia Ribeiro^1,2#^, Inês Delgado^1^, Bárbara Peleteiro^1,2^, Martine Aggerbeck^3,4^, Emilie Distel^3,4^, Isabella Annesi-Maesano^5^, Denis Sarigiannis^6^, Elisabete Ramos^1,2*^


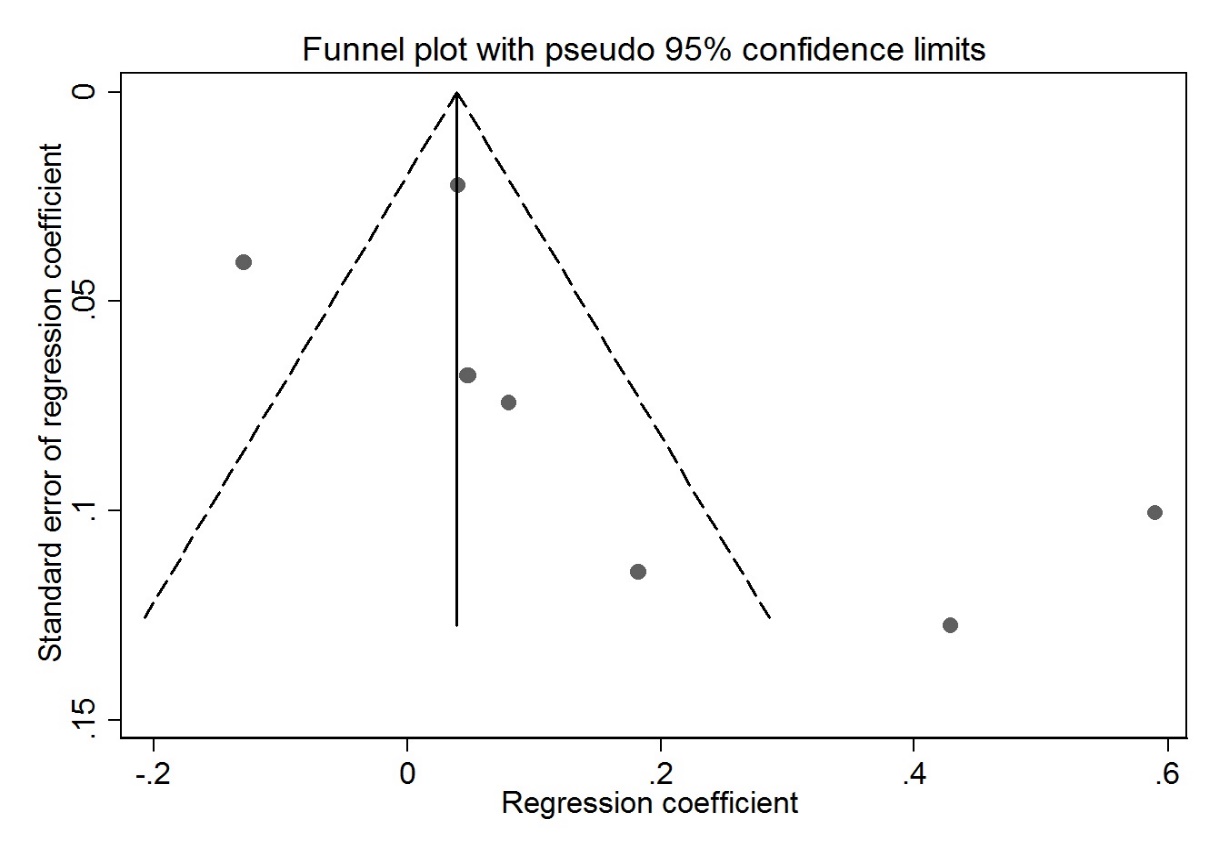


**Supplemntary Figure S1.** Funnel plot of publications assessing the relation between oxychlordane and (A) body mass index and (B) diabetes-related features

**A**

**B**


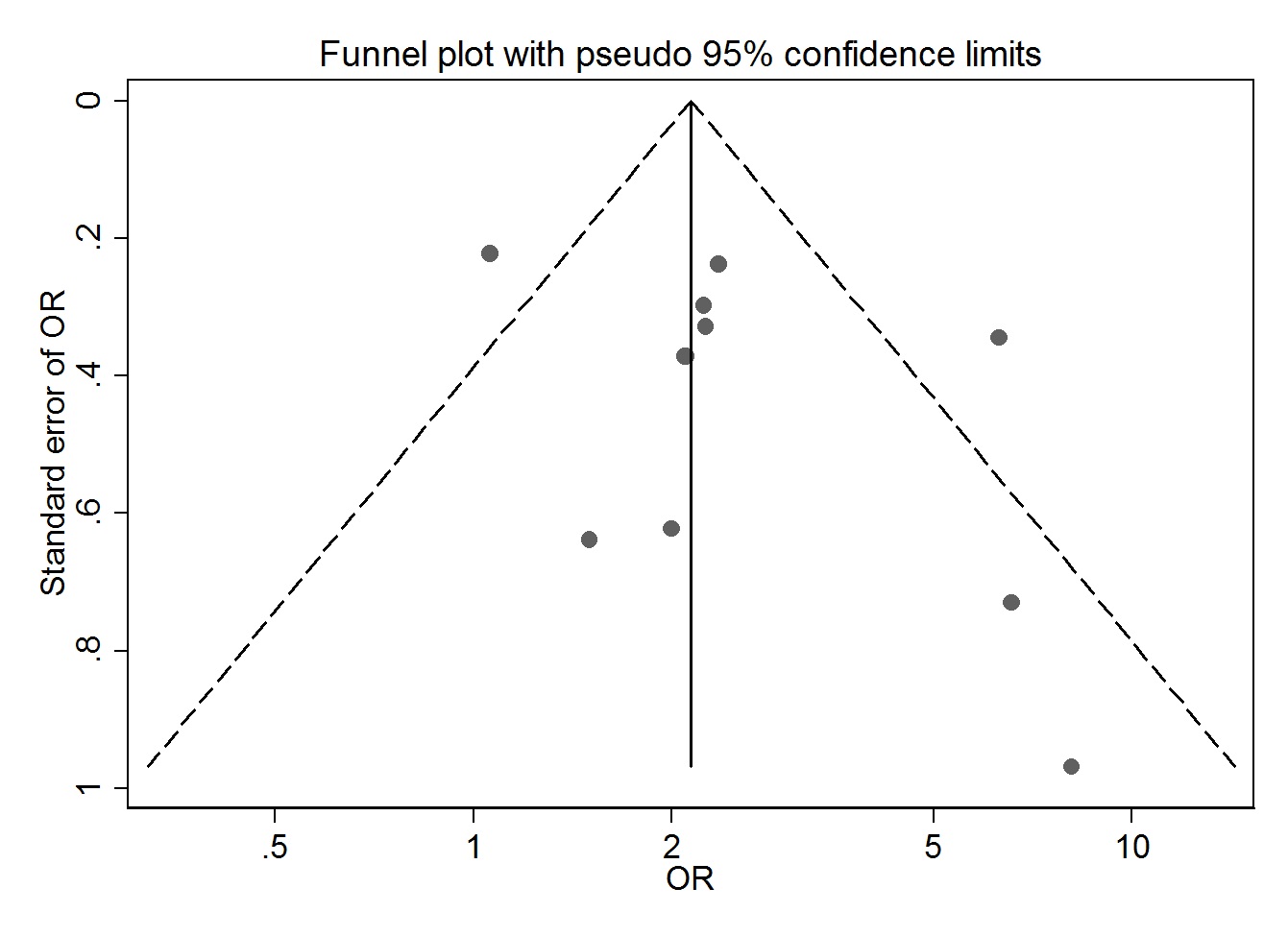

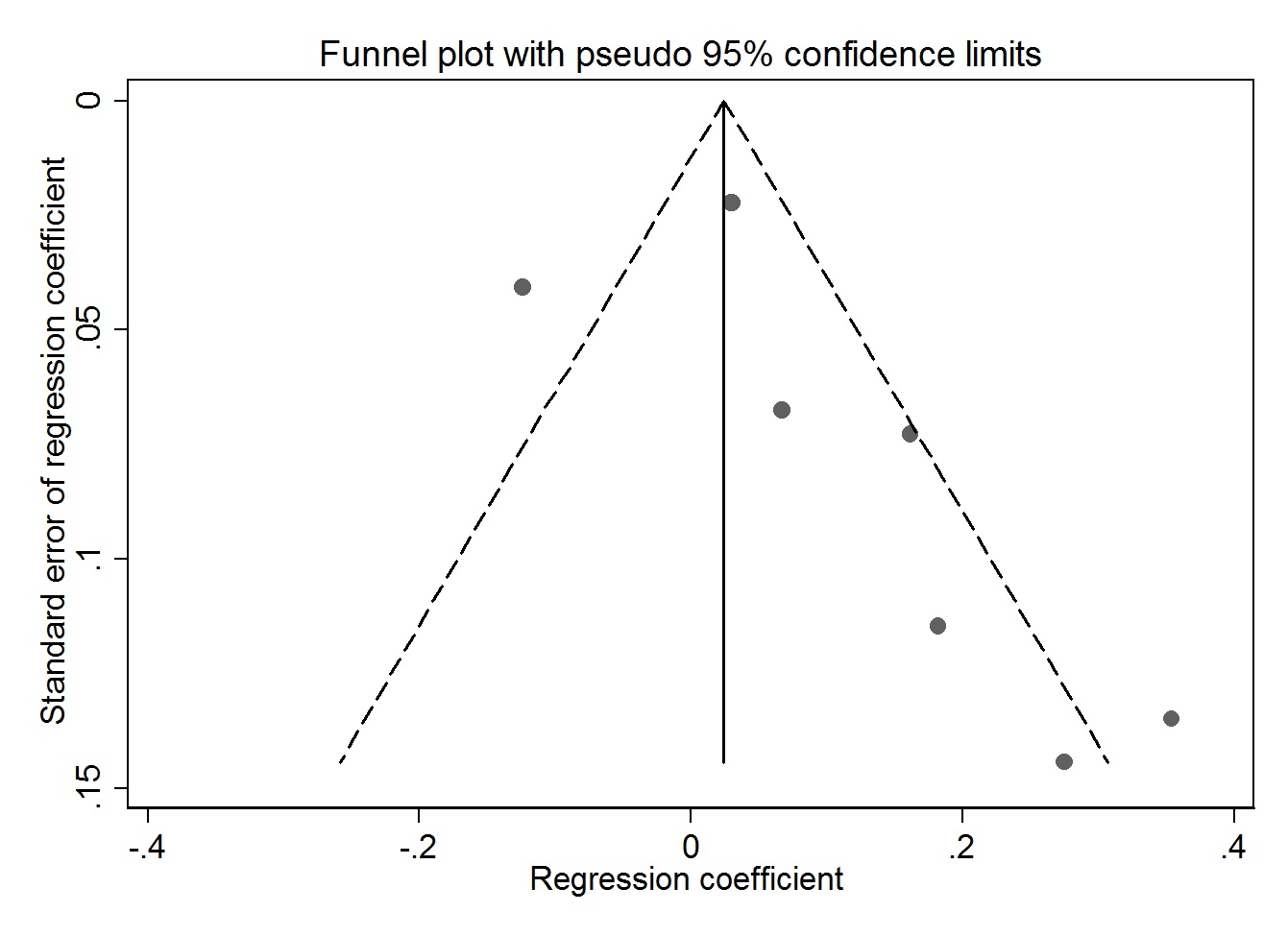


**Supplemntary Figure S2.** Funnel plot of publications assessing the relation between trans-nonachlor and (A) body mass index and (B) diabetes-related features.

**A**

**B**

**Supplemntary Figure S3.** Funnel plot of papers assessing the relation between heptachlor epoxide and diabetes-related features

**Supplementary Data S1.** Potential Mechanism of Action of chlordane effects on diabetes

A PubMed search with the keywords (chlordane OR oxychlordane OR nonachlor OR heptachlor OR heptachlor epoxide) AND either gene expression OR molecular mechanism OR nuclear receptor retrieved 93 independent publications and the keywords (chlordane OR oxychlordane OR nonachlor OR heptachlor OR heptachlor epoxide) AND either constitutive androstane receptor (CAR) OR pregnane X receptor (PXR) retrieved only 5 additional publications. The addition of the words diabetes or adiposity or obesity did not retrieve any additional publications. After careful reading of either the abstract or the entire article, we retained only 25 articles, which include experiments to elucidate molecular mechanisms of action mainly in human or rodent models (supplementary Table S5). *In vitro* studies showed that members of the chlordane family may act via multiple signaling pathways, including the activation of several nuclear receptors. Using human reporter cell lines and binding assays, chlordanes were described as low affinity ligands of the retinoic acid receptors (RARβ and RARγ) [1] and as agonists of the estrogen receptors (ERα and ERβ) [2, 3]. Chlordane also was described as an antagonist of the estrogen receptor-related receptor ERRα1 [4-7]. Chlordane and trans-nonachlor also can activate the PXR [8, 9]. In the human liver-derived HepaRG cell line, chlordane, as well as other organochloride pesticides, were found to modify the expressions and activities of several drug transporters via PXR [10]. In human THP1 monocytes, trans-nonachlor also was shown to induce the production of the superoxide anion via NADPH oxidase leading, potentially, to pathogenic oxidative stress, inflammation and atherosclerosis [11, 12]. Exposure of the mouse NIH3T3-L1 adipocyte cell line to oxychlordane prior to and during differentiation did not modify adipogenesis whereas it increased basal fatty acid uptake in differentiated adipocytes, which may cause adipocyte hypertrophy, without modification of lipolysis [13]. Chlordane and trans-nonachlor were also able to disrupt epidermal growth factor (EGF) receptor signaling in the hepatic HepG2 cells and in the epidermoid carcinoma A-431 cells by displacing EGF from its receptor in the extracellular domain [14]. Finally, although the exact mechanism is not deciphered, heptachlor decreases neurite length in a dose-dependent manner in the rat PC-12 cell model [15] following overexpression of the Gap-43 protein. *In vivo*, in wild-type, humanized CAR/PXR mice and CAR/PXR knock-out (KO) animals, chlordane induced liver hypertrophy and hyperplasia via the CAR and PXR pathways. Gene expression of Cyp2B10 and Cyp3A11, two known targets of CAR, was increased irrespective of the species from which the receptors were derived whereas only mouse receptors were found to increase liver hyperplasia [16]. These results confirm the marked species differences between mice and humans for PXR and CAR activation [17]. Moreover, a recent analysis of the mode of action (MOA) of another CAR agonist, phenobarbital, concluded that the MOA in rodents does not support a plausible MOA for humans [18]. To alleviate the species differences, a recent study using wild-type mice, CAR/PXR double KO and CAR/PXR double humanized mice has shown that the hepatic dlk1-Dio3 non-coding RNA locus is activated by chlordane via CAR and could be used as an early biomarker locus for nongenotoxic hepatocarcinogenesis [19]. A recent metabolomic study of the effects of technical chlordane together with a high fat diet (HFD) in CD-1 mice suggested that chlordane and HFD as well as their combination profoundly modified tryptophan metabolism, altered the homeostasis of gut microorganisms and disturbed the energy metabolism of the mice (glucose, glycogen and tricarboxylic acid cycle) [20]. *In utero* exposure of mice to heptachlor was found to increase dopamine transporter levels in the brain probably via the increased expression of the nuclear transcription factor Nurr-1 (Nuclear receptor related-1) [21]. This could alter dopamine availability for other organs, with a greater susceptibility of males to the pollutant. In particular, dopamine reduces insulin production in the pancreas [22] and, therefore, alterations of dopamine levels by members of the chlordane family could play a role in the development of diabetes. In a different animal model, the polar bear, oxychlordane was described as a weak antagonist of the bear peroxisome proliferator-activated receptor gamma (PPARg) in a luciferase construct in cos7 cells [23]. Since PPARg plays an important role in adipogenesis, compounds such as chlordanes could hamper the formation of lipid stores in this species. Finally, an epidemiological study has shown an increased level of several organochlorine pesticides, among which heptachlor, in patients with epithelial ovarian cancer compared to control ones. A transcriptomic study comparing gene expression in the ovary tissues from the two groups reveals that several pathways are dysregulated, especially cytoskeleton remodeling but also the bone morphogenetic protein 7 (BMP7) pathway [24], through the TGF-WNT signaling pathway. BMP7 plays a role in brown adipogenesis and energy expenditure [25] and has been described as regulated by another pollutant, bisphenol A [26].

All these data highlight the fact that chlordanes activate several nuclear receptors, in particular ER and PXR, whereas they antagonize ERRα1. However, the molecular mechanism(s) to explain the epidemiological association between exposure to these organochlorine compounds and diabetes/adiposity has not been studied extensively in human models. Recently, it has been suggested that ERs [27, 28] as well as the ERR [29-31] are involved in type 2 diabetes. With respect to PXR, one study conducted in twelve healthy volunteers found that administration of the human PXR agonist, rifampicin (also known as rifampin) elicited postprandial hyperglycemia and, thus, could be detrimental to glucose tolerance [32]. Moreover, PXR ablation (KO) in both wild type and *ob/ob* mice inhibited HFD-induced obesity and insulin resistance [33] whereas CAR ablation worsened insulin insensitivity [34]. However, it should be kept in mind that there are marked species differences between mice and humans for PXR and CAR activation. Moreover, new possible players have been recently described and may be involved also in the effect of chlordanes.

It is important to highlight that research on the health effects of EDCs in humans is a challenging field, since individuals are often exposed to a wide mixture of substances. EDCs levels of exposure are generally highly correlated with each other, limiting the ability to estimate the impact of a sole chemical. On the other hand, these correlations are not perfect and an individual who has low levels of one EDC may have high levels of another which may lead to over- or under-estimation of the associations [3].

**Supplementary Table S1:** Quality assessment of reporting of the epidemiological studies included in the meta-analyses for the association between chlordanes, adiposity and diabetes-related features

| **Reference** | **Total** |
| --- | --- |
| Pelletier, Cet al. (2002) | 13.60 |
| Magnusdottir, E et al. (2005) | 15.22 |
| Lee, DH et al. (2006) | 15.43 |
| Hue, O et al. (2006) | 13.76 |
| Cox et al. (2007) | 15.5 |
| Everett et al. (2010) | 15.25 |
| Lee et al. (2010) | 18.45 |
| Son, HK et al. (2010) | 18.9 |
| Airaksinen et al. (2011) | 18.00 |
| Lee et al. (2011) | 18.00 |
| Rylander, L et al. (2015) | 19.30 |
| Eden, PR et al. (2016) | 18.00 |
| Jaacks, LM et al. (2016) | 17.49 |
| Han, et al (2020) | ~~18.45~~ |
| Grice, BA et al. (2017) | 14.75 |
| Rosenbaum, PF et al. (2017) | 17.92 |
| Kim, JT et al. (2018) | 17.06 |

**Supplementary Table S2:** Description of studies’ characteristics

| **Adiposity** | | | | | | | |
| --- | --- | --- | --- | --- | --- | --- | --- |
| **Authors, year of publication** | **Country** | **Type of study** | **Subjects characteristics** | **Age group** | **Exposure Assessment** | **Outcome Assessment** | **Confounders** |
| Pelletier, C et al. (2002) | CAN | CS | Lean sedentary and obese men;  ♂n=43 | Adults  (36-58) | Serum  (GC) | - | Age |
| Magnusdottir, E et al. (2005) | ISL | CS | Infertile men;  ♂ n=72 | Adults (37±5.4)* | Serum  (GC) | Self-reported W&H | - |
| Hue, O et al. (2006) | CAN | CS | Obese selected for BPD-DS and NW;  ♂ n=42 | Adults | Serum  (GC-DE) | BMI, WC (Measured) | - |
| Lee, DH et al. (2006) | USA | CS | NHANES  1999-2002;  ♂ n= 903  ♀ n= 1113 | Adults (≥20) | Serum (HRGC/  HRMS) | Measured W&H | - |
| Lee, DH et al. (2007b) | USA | CS | NHANES  1999-2002;  Non-diabetic;  ♂♀ n= 721 | Adults (≥20) | Serum  (HRGC/  IDHRMS) | WC (Measured)  ♂>102 cm,  ♀>88 cm | Age, sex, race, PIR, smoking, alcohol, exercise |
| Cho, MR et al. (2011) | USA | CS | NHANES,  1999-2004;  ♂ n=768; <50y.o  ♂ n=612; ≥50y.o  ♀ n=710; <50y.o  ♀ n=679; ≥50y.o | Adults | Serum  (HRGC/  IDHRMS) | DXA | Age, race, PIR, smoking, PA, height |
| Ronn, M et al. (2011) | SWE | PC | PIVUS;  ♂ n=431  ♀ n=459 | Adults  (70y.o.) | Serum  (HRGC/  HRMS) | FM (DXA) | - |
| Lee, DH et al. (2012) | SWE | CS | PIVUS,  2001-2004;  ♂ n=490  ♀ n=480 | Adults  (70y.o.) | Plasma  (HRGC/  HRMS) | WC (Measured) ♂>102cm | Calories, exercise, smoking, TG, TC, alcohol |
| Lee, DH et al. (2012) | SWE | PC | PIVUS,  2001-2009;  ♂ n=305  ♀ n= 206 | Adults (≥75) | Plasma  (HRGC  /HRMS) | WC (Measured) ♂>102cm | Calories, exercise, smoking, TG, TC, alcohol |
| Roos, V et al. (2013) | SWE | CS | PIVUS;  ♂♀n=287 | Adults  (70y.o.) | Plasma  (HRGC/  HRMS) | VAT (MRI)  SAT (MRI) | Gender, education, PA and smoking |
| Jaacks, LM et al. (2016) | USA | CS | LIFE study;  ♀ n= 218 | Adults  (18-40) | Serum | Measured W&H | - |
| Bjerregaard-Olesen, C et al. (2017) | DEN | CS | Pregnant women;  ♀ n= 218 | Adults  (26-32) | Serum  (GC-MS) | Pre-pregnancy BMI (Self-reported W&H) | Age, smoking |
| Rosenbaum, PF et al. (2017) | USA | CS | ACHS;  ♂♀ n=584 | Adults  (53.6± 16.2)* | Serum  (HRGC/  ID-HRMS) | Measured W&H | - |
| Kim, JT et al. (2018) | KOR | CS | General pop.;  ♂♀ n=180 | Adults  (41-69) | Serum  (HRMS/GC) | Self-reported W&H | - |
| Chen MW et al (2018) | TWN | CS | Pregnant woman  ♀ n=68 | Adults (19-40) | Beast Milk  (HRGC/LRMS) | Pre-pregnancy BMI (self-reported W&H) | age, population, annual income, birth year and parity |
| Lauritzen HB et al. (2016) | NOR  SWE | PC | Pregnant woman  ♀ n=424 | Adults (18-41) | Seum  (GC-MS) | Pre-pregnancy BMI (self-reported W&H) | Study site (NOR/SWE), maternal age, education,  height, smoking, alcohol, parity, previous BF duration and time since end of last BF period |
| **Diabetes-related features** | | | | | | | |
| **Authors, year of publication** | **Country** | **Type of study** | **Subjects characteristics** | **Age group** | **Exposure Assessment** | **Outcome Assessment** | **Confounders** |
| Lee, DH et al. (2006) | USA | CS | NHANES  1999-2002;  ♂n= 903, ♀n=1113 | Adults  (≥20) | Total blood (HRGC/  HRMS) | FBG≥ 126mg/dL or NFBG≥200 mg/dL or  Self-reported | Age, sex, race, PIR, BMI, WC |
| Cox et al. (2007) | USA | CS | HHANES  1982-1984;  ♂♀ n= 1303 | Adults  (20-74) | Serum  (GC) | Self-reported | Age, BMI |
| Lee, DH et al. (2007a) | USA | CS | NHANES  Non-diabetic  1999-2002;  ♂n= 347, ♀n=402 | Adults  (20-85) | Serum (HRGC/  HRMS) | HOMA-IR  ≥5.06 mU/lmmol/L | Age, sex, race, PIR, BMI, WC, smoking, alcohol, exercise |
| Lee, DH et al. (2007b) | USA | CS | NHANES  Non-diabetic  1999-2002;  ♂♀ n= 721 | Adults  (≥20) | Total blood  (HRGC/  HRMS) | FBG  ≥126mg/dL | Age, sex, race, PIR, smoking, alcohol, exercise |
| Everett, CJ et al. (2010) | USA | CS | NHANES  1999-2004;  ♂♀ n= 281 | Adults  (≥20) | Serum  (GC-IDHRMS) | HbA1c  5.7-6.4% | Age, gender, race, education, PIR, BMI, WC, PA, family history DM |
| Lee, DH et al (2010) | USA | NCC | CARDIA  1987-2006 | Adults  (20-36) | Serum  (GC-IDHRMS) | FBG, Med | age, sex, race, and BMI at year2, triglyceride and TC at year 2 |
| Park, SK et al. (2010) | KOR | CS | Individuals w/ metabolic syndrome;  ♂n=14,♀n=36 | Adults  (56.5±  6.9)* | Serum  (GC-IDHRMS) | HOMA-IR: FBG(mmol/L) x FBI(mU/L)/  22.5 | Age, sex, alcohol, smoking, BMI |
| Park, SK et al. (2010) | KOR | CS | Individuals w/o metabolic syndrome; ♂n=14, ♀n=36 | Adults  (56.5±  6.9)* | Serum  (GC-IDHRMS) | HOMA-IR: FBG(mmol/L) x FBI(mU/L)/  22.5 | Age, sex, alcohol, smoking, BMI |
| Patel, CJ et al. (2010) | USA | CS | NHANES  1999-2004;  ♂♀ n=2545 | Adults | Total blood^†^ | FBG  ≥126 mg/dL | Age, sex, BMI, ethnicity, SES |
| Son, et al. (2010) | KOR | CC; | Diabetics and controls  ♂♀ n= 80 | Adults | Serum  (IDHRMS) | FBG, Med | Age, sex, body mass index, alcohol and smoking |
| Airaksinen, et al. (2011) | FIN | CS | HBCS 2003  ♂♀ n= 1988 | Adults (62)* | Serum  (GC) | FBG, Med, OGTT | - |
| Lee, DH et al. (2011) | SWE | PC | PIVUS,  2001-2009;  ♂ n= 350  ♀ n= 375 | Adults  (≥70) | Total blood  (HRGC/  HRMS) | FBG≥  112mg/dL or medication | Sex, BMI, smoking, exercise, TG, TC, alcohol |
| Starling, AP et al. (2014) | USA | PC | Spouses of pesticide applicators; ♀ n=13637 | Adults (17-88) | Self-reported (pesticide use) | Self-reported | Age, residence, BMI |
| Rylander et al. (2015) | NOR | PC | NORWAC  ♀ n= 212 | Adults  (30-70) | Serum  (GC–MS) | Self-reported | - |
| Eden, PR et al. (2016) | USA | CS | Military and families  ♂♀ n= 263 | Adults  (18-65) | Serum  (IDHRMS) | Self-reported, Med records | - |
| Zong, G et al. (2016) | USA | CS | NHANES  ♀ n= 1165 | Adults | Serum  (GC-IDHRMS) | Self-reported, Med, HbA1c ≥6.5% | age, survey years, ethnicity, country of born, education, and others, |
| Grice, BA et al. (2017) | USA | NCC | General population without diabetes  ♂♀ n= 300 | Adults | Serum  (GC-IDHRMS) | OGTT | age, sex, BMI, 2hPG, sample water loss, storage time, TC and triglycerides |
| Han, X., et al. (2020) | CHN | CC | General population  with DM n=265  without DM n=242 ♂♀ | Adults  (25-74) | Serum  (HRGC/HRMS) | FBG. Med | age, sex, BMI, TC and triglycerides |
| Cordier et al. (2020) | CAN | CS | Indigenous population  Cree ♂♀ (n=778)  Inuit ♂♀ (n=877) | Adult  (33.8)*  (32.9)* | Serum  SPE-HRCG-MS | Medical diagnose or med use | Age, sex, WC, smoking, omega-3 PUFAs, total lipids |
| **CS**: Cross-sectional; **CC**: case control; **PC**: Prospective Cohort; **NCC**: Nested Case Control; **NW**: Normal weight; **BDL**: Below Detected Levels; * Mean; **y.o.:** years old; **NHANES**: National Health and Nutrition Examination Survey; **PIVUS**: Prospective Investigation of the Vasculature in Uppsala Seniors; **ACHS**: Anniston Community Health Survey; **LIFE study:** Longitudinal Investigation of Fertility and the Environment study; **GC**: Gas chromatography; **HBCS:** Helsinki Birth Cohort Study; **CARDIA**: Coronary Artery Risk Development in Young Adults; **NORWAC**: Norwegian Women and Cancer study; **HRGC**: High Resolution Gas Chromatography; **HRMS**: High Resolution Mass Spectrometry **IDHRMS**: Isotope Dilution High Resolution Mass Spectrometry; **MS**: Mass Spectrometry; **LRMS**: Low resolution mass spectrometry; **MRI**: Magnetic Resonance Imaging; **DXA**: Dual-energy X-ray absorptiometry; **FM**: Fat Mass; **BMI**: Body Mass Index; **WC**: waist circumference; **VAT**: Visceral adipose tissue; **SAT**: Subcutaneous adipose tissue; **W&H:** Weight and height; **FBG:** fasting blood glucose;  **NFBG:**  nonfasting blood glucose; **HOMA-IR**: Homeostatic model assessment - insulin resistance; **Hb1A1c**: hemoglobin A1c; **OGTT:** glucose tolerance test; **IR**: Insulin Resistance; **FBI**: Fasting Blood Insulin **Med**: Medication; **DM**: Diabetes Mellitus; **PA**: Physical Activity; **PIR**: Poverty Income Ratio; **TG**: triglycerides; **TC**: Total Cholesterol; **SES**: Socioeconomic; **BF**: Breastfeeding Status †No further information | | | | | | | |

| **Supplementary Table S3.** Summary estimates for the association between oxychlordane and each adiposity measure | | | | |
| --- | --- | --- | --- | --- |
|  | **Number of included estimates** | **Summary Estimate*** | **I^2^ (%)** | **95% CI** |
|  | **Oxychlordane** | | | |
|  |  |  |  |  |
| **Body Mass Index** | 8^a^ | 0.01 | 88.6 | -0.01; 0.03 |
| **Fat Mass** | 5^b^ | 0.03 | 90.0 | -0.01; 0.07 |
| **Waist Circumference** | 1^c^ | 0.04^†^ | - | >0.01^‡^ |
| **Diabetes-related features** | 8^d^ | 1.96 | 69.8 | 1.19; 3.23 |
|  | **Trans-nonachlor** | | | |
|  |  |  |  |  |
| **Body Mass Index** | 9^e^ | -0.01 | 75.7 | -0.03; 0.01 |
| **Fat Mass** | 5^f^ | 0.01 | 81.7 | -0.03; 0.04 |
| **Waist Circumference** | 1^g^ | 0.01^†^ | - | >0.01^‡^ |
| **Visceral Adipose Tissue** | 1^h^ | 17.0 | - | 4.7; 30.0 |
| **Subcutaneous Adipose Tissue** | 1^i^ | 19.0 | - | -2.6; 40.0 |
| **Diabetes-related features** | 9^j^ | 2.04 | 41.1 | 1.47;2.85 |
|  | **Heptachlor** **Epoxide** | | | |
|  |  |  |  |  |
| **Diabetes-related features** | 3^k^ | 1.88 | 0.00 | 1.42; 2.49 |
| I^2^ available only when meta-analysis was performed.  *Beta coefficient if not otherwise specified; ^†^Spearman correlation coefficient; ^‡^p value.  **Oxychlordane** Included studies are: ^a^Pelletier, C et al. (2002), Magnusdottir, EV et al. (2005), Hue, O et al. (2006), Lee, DH et al. (2006), Jaacks, LM et al. (2016) , Bjerregaard-Olesen, C et al. (2017) , Rosenbaum, PF et al. (2017) , Kim, JT et al. (2018) ; ^b^Pelletier, C et al. (2002) and Cho, MR et al. (2011) ^c^Lee, DH et al. (2006). ^d^ Airaksinen et al. (2010); Cox, et al. (2007); Eden, et al. (2016); Everett, et al. (2010); Grice, et al. (2017) Lee et al. (2006); Rylander et al. (2015); Son (2010) **Trans-nonachlor** Included studies are: ^e^Pelletier, C et al. (2002), Magnusdottir, EV et al. (2005), Hue, O et al. (2006) and Lee, DH et al. (2006) Jaacks, LM et al. (2016) Bjerregaard-Olesen, C et al. (2017) Rosebaum (2017) Kim, JT et al. (2018); ^f^ Pelletier, C et al. (2002) and Cho, MR et al. (2011); ^g^ Lee, DH et al. (2006); ^h^Roos, V et al. (2013); ^i^Roos, V et al. (2013); ^j^ Airaksinen et al. (2010) Cox, et al. (2007); Eden, et al. (2016); Everett, et al. (2010); Grice, et al. (2017) Lee et al. (2010); Lee et al. (2011); Rylander et al. (2015); Son (2010).**Heptachlor Epoxide** Included studies are ^k^ Everett, et al. (2010) ; Son (2010); Starling, et al. (2014) | | | | |

| **Supplementary Table S4.** Description of the only paper assessing the association between the sum of chlordanes (oxychlordane and trans-chlordane) and diabetes-related features. | | | | | | | | | |
| --- | --- | --- | --- | --- | --- | --- | --- | --- | --- |
| **Authors, year of publication** | **Country** | **Type of study** | **Subjects characteristics** | **Age group** | **Exposure Assessment** | **Outcome Assessment** | **Exposure contrast** | **Estimate (95% CI)** | **Adjustment for confounders** |
| \| **Diabetes-related features** \| \| \| \| \| \|  \|  \|  \|  \| \| --- \| --- \| --- \| --- \| --- \| --- \| --- \| --- \| --- \| --- \| \|  \|  \|  \|  \| \| Cordier et al. (2020) \| CAN \| CS \| Indigenous population Inuit ♂♀ (n=877) \| Adult (32.9)* \| SPE-HRCG-MS \| Medical diagnose dor med use \| ng/g lipids \| OR=2.11 (0.7; 6.36) \| Age, sex, WC, smoking, omega-3 PUFAs, total lipids \| \| Cordier et al. (2020) \| CAN \| CS \| Indigenous population Cree ♂♀ (n=778) \| Adult  (33.8)* \| SPE-HRCG-MS \| Medical diagnose dor med use \| ng/g lipids \| OR=5.83 (2.58; 13.2) \| Age, sex, WC, smoking, omega-3 PUFAs, total lipids \| | | | | | | | | | |
| **CS**: Cross-sectional; *Mean; **HRGC**: High Resolution Gas Chromatography **MS**: Mass Spectrometry; **WC**: waist circumference **OR**: Odds Ratio. | | | | | | | | | |

**Supplementary Table S5.** Molecular signaling pathways implicated in the effects of the chlordane compounds

| **Compound** | **Model (cell line/animal/other)** | **Molecular/signaling pathway** | **Reference** |
| --- | --- | --- | --- |
| Chlordane | HepG2 (h), cos 7 (mo) | RARβ and RARγ | [1] |
| Chlordane, heptachlor, trans-nonachlor | HELN (h) | ERα and ERβ | [2] |
| Chlordane | MCF7 (h), Hepa1c1c7 (m) | ER | [35] |
| Heptachlor | MCF10AT (h), MCF7 (h) | ER | [36] |
| Chlordane | T47D (h) | ER | [7] |
| Chlordane, heptachlor | CHO-K1 cells (ch) transfected with various nuclear receptor reporter constructs | ER | [3] |
| Trans-nonachlor | Recombinant proteins (h) in E Coli | ER/PR | [37] |
| Chlordane, heptachlor | Rat liver | ER/AR | [38] |
| Chlordane | WPMY-1 (h) | ERRα1 | [6] |
| Chlordane | SK-BR-3 (h) | ERRα1 | [5] |
| Chlordane | SK-BR-3 (h), yeast assay | ERRα1 | [4] |
| Chlordane, trans-nonachlor | LLC-PK1 (p) | PXR | [39] |
| Chlordane, trans-nonachlor | HG5LN (h), HepG2 (h), LS174T (h) | PXR | [40] |
| Trans-nonachlor | HuH7 (h) | PXR | [9] |
| Chlordane | HepG2 (h), MCF7 (h) | PXR | [8] |
| Wild-type, KO and humanized mice |  | PXR and CAR | [16, 19] |
| Trans-nonachlor | THP1 (h) | Oxidative stress | [11] |
| Heptachlor | SH-SY5Y (h) | Oxidative stress | [41] |
| Heptachlor epoxide | Hepa1c1c7 (m) | Ca2+, PLCγ1, AP1 | [42] |
| Heptachlor | Lymphocytes (h) | MAPK | [43] |
| Oxychlordane | Cos 7 cells (mo) transfected with bear-PPARg reporter constructs | PPARg | [23] |
| Chlordane, trans-nonachlor | HepG2 (h), A-431 (h) | EGFR | [14] |
| Heptachlor | PC12 (r) | Neurite length through gap-43 overexpression | [15] |
| Heptachlor, Heptachlor epoxide B | Human ovarian cancer tissues | TGF, WNT | [24] |

**References**

1. Lemaire, G., et al., *Activation of retinoic acid receptor-dependent transcription by organochlorine pesticides.* Toxicol Appl Pharmacol, 2005. **202**(1): p. 38-49.

2. Lemaire, G., et al., *Activation of alpha- and beta-estrogen receptors by persistent pesticides in reporter cell lines.* Life Sci, 2006. **79**(12): p. 1160-9.

3. Zhang, J., et al., *Determination of endocrine-disrupting potencies of agricultural soils in China via a battery of steroid receptor bioassays.* Environ Pollut, 2018. **234**: p. 846-854.

4. Chen, S., et al., *Modulation of aromatase expression in human breast tissue.* J Steroid Biochem Mol Biol, 2001. **79**(1-5): p. 35-40.

5. Yang, C. and S. Chen, *Two organochlorine pesticides, toxaphene and chlordane, are antagonists for estrogen-related receptor alpha-1 orphan receptor.* Cancer Res, 1999. **59**(18): p. 4519-24.

6. Miao, L., et al., *Estrogen receptor-related receptor alpha mediates up-regulation of aromatase expression by prostaglandin E2 in prostate stromal cells.* Mol Endocrinol, 2010. **24**(6): p. 1175-86.

7. Houtman, C.J., et al., *Biological validation of a sample preparation method for ER-CALUX bioanalysis of estrogenic activity in sediment using mixtures of xeno-estrogens.* Environ Sci Technol, 2006. **40**(7): p. 2455-61.

8. Coumoul, X., M. Diry, and R. Barouki, *PXR-dependent induction of human CYP3A4 gene expression by organochlorine pesticides.* Biochem Pharmacol, 2002. **64**(10): p. 1513-9.

9. Jacobs, M.N., G.T. Nolan, and S.R. Hood, *Lignans, bacteriocides and organochlorine compounds activate the human pregnane X receptor (PXR).* Toxicol Appl Pharmacol, 2005. **209**(2): p. 123-33.

10. Bucher, S., et al., *Regulation of hepatic drug transporter activity and expression by organochlorine pesticides.* J Biochem Mol Toxicol, 2014. **28**(3): p. 119-28.

11. Mangum, L.C., et al., *The association of serum trans-nonachlor levels with atherosclerosis.* J Toxicol Environ Health A, 2016. **79**(5): p. 210-20.

12. Mangum, L.C., et al., *Organochlorine insecticides induce NADPH oxidase-dependent reactive oxygen species in human monocytic cells via phospholipase A2/arachidonic acid.* Chem Res Toxicol, 2015. **28**(4): p. 570-84.

13. Howell, G., 3rd and L. Mangum, *Exposure to bioaccumulative organochlorine compounds alters adipogenesis, fatty acid uptake, and adipokine production in NIH3T3-L1 cells.* Toxicol In Vitro, 2011. **25**(1): p. 394-402.

14. Hardesty, J.E., et al., *Epidermal Growth Factor Receptor Signaling Disruption by Endocrine and Metabolic Disrupting Chemicals.* Toxicol Sci, 2018. **162**(2): p. 622-634.

15. Christen, V., et al., *Developmental neurotoxicity of different pesticides in PC-12 cells in vitro.* Toxicol Appl Pharmacol, 2017. **325**: p. 25-36.

16. Ross, J., et al., *Human constitutive androstane receptor (CAR) and pregnane X receptor (PXR) support the hypertrophic but not the hyperplastic response to the murine nongenotoxic hepatocarcinogens phenobarbital and chlordane in vivo.* Toxicol Sci, 2010. **116**(2): p. 452-66.

17. Cheung, C. and F.J. Gonzalez, *Humanized mouse lines and their application for prediction of human drug metabolism and toxicological risk assessment.* J Pharmacol Exp Ther, 2008. **327**(2): p. 288-99.

18. Elcombe, C.R., et al., *Mode of action and human relevance analysis for nuclear receptor-mediated liver toxicity: A case study with phenobarbital as a model constitutive androstane receptor (CAR) activator.* Crit Rev Toxicol, 2014. **44**(1): p. 64-82.

19. Pouche, L., et al., *Xenobiotic CAR Activators Induce Dlk1-Dio3 Locus Noncoding RNA Expression in Mouse Liver.* Toxicol Sci, 2017. **158**(2): p. 367-378.

20. Wang, D., et al., *The fate of technical-grade chlordane in mice fed a high-fat diet and its roles as a candidate obesogen.* Environ Pollut, 2017. **222**: p. 532-542.

21. Richardson, J.R., et al., *Developmental heptachlor exposure increases susceptibility of dopamine neurons to N-methyl-4-phenyl-1,2,3,6-tetrahydropyridine (MPTP)in a gender-specific manner.* Neurotoxicology, 2008. **29**(5): p. 855-63.

22. Ustione, A., D.W. Piston, and P.E. Harris, *Minireview: Dopaminergic regulation of insulin secretion from the pancreatic islet.* Mol Endocrinol, 2013. **27**(8): p. 1198-207.

23. Routti, H., et al., *Environmental Chemicals Modulate Polar Bear (Ursus maritimus) Peroxisome Proliferator-Activated Receptor Gamma (PPARG) and Adipogenesis in Vitro.* Environ Sci Technol, 2016. **50**(19): p. 10708-10720.

24. Shah, H.K., et al., *Delineating Potential Transcriptomic Association with Organochlorine Pesticides in the Etiology of Epithelial Ovarian Cancer.* Open Biochem J, 2018. **12**: p. 16-28.

25. Tseng, Y.H., et al., *New role of bone morphogenetic protein 7 in brown adipogenesis and energy expenditure.* Nature, 2008. **454**(7207): p. 1000-4.

26. Kusumegi, T., et al., *BMP7/ActRIIB regulates estrogen-dependent apoptosis: new biomarkers for environmental estrogens.* J Biochem Mol Toxicol, 2004. **18**(1): p. 1-11.

27. Barros, R.P., U.F. Machado, and J.A. Gustafsson, *Estrogen receptors: new players in diabetes mellitus.* Trends Mol Med, 2006. **12**(9): p. 425-31.

28. Hevener, A.L., D.J. Clegg, and F. Mauvais-Jarvis, *Impaired estrogen receptor action in the pathogenesis of the metabolic syndrome.* Mol Cell Endocrinol, 2015. **418 Pt 3**: p. 306-21.

29. Mootha, V.K., et al., *Erralpha and Gabpa/b specify PGC-1alpha-dependent oxidative phosphorylation gene expression that is altered in diabetic muscle.* Proc Natl Acad Sci U S A, 2004. **101**(17): p. 6570-5.

30. Huss, J.M., W.G. Garbacz, and W. Xie, *Constitutive activities of estrogen-related receptors: Transcriptional regulation of metabolism by the ERR pathways in health and disease.* Biochim Biophys Acta, 2015. **1852**(9): p. 1912-27.

31. Audet-Walsh, E. and V. Giguere, *The multiple universes of estrogen-related receptor alpha and gamma in metabolic control and related diseases.* Acta Pharmacol Sin, 2015. **36**(1): p. 51-61.

32. Rysa, J., et al., *Pregnane X receptor agonists impair postprandial glucose tolerance.* Clin Pharmacol Ther, 2013. **93**(6): p. 556-63.

33. He, J., et al., *PXR ablation alleviates diet-induced and genetic obesity and insulin resistance in mice.* Diabetes, 2013. **62**(6): p. 1876-87.

34. Gao, J., et al., *The constitutive androstane receptor is an anti-obesity nuclear receptor that improves insulin sensitivity.* J Biol Chem, 2009. **284**(38): p. 25984-92.

35. Legler, J., et al., *Development of a stably transfected estrogen receptor-mediated luciferase reporter gene assay in the human T47D breast cancer cell line.* Toxicol Sci, 1999. **48**(1): p. 55-66.

36. Shekhar, P.V., J. Werdell, and V.S. Basrur, *Environmental estrogen stimulation of growth and estrogen receptor function in preneoplastic and cancerous human breast cell lines.* J Natl Cancer Inst, 1997. **89**(23): p. 1774-82.

37. Scippo, M.L., et al., *Recombinant human estrogen, androgen and progesterone receptors for detection of potential endocrine disruptors.* Anal Bioanal Chem, 2004. **378**(3): p. 664-9.

38. Jung, K.H., et al., *Characteristic molecular signature for early detection and prediction of persistent organic pollutants in rat liver.* Environ Sci Technol, 2012. **46**(23): p. 12882-9.

39. Schuetz, E.G., C. Brimer, and J.D. Schuetz, *Environmental xenobiotics and the antihormones cyproterone acetate and spironolactone use the nuclear hormone pregnenolone X receptor to activate the CYP3A23 hormone response element.* Mol Pharmacol, 1998. **54**(6): p. 1113-7.

40. Delfosse, V., et al., *Synergistic activation of human pregnane X receptor by binary cocktails of pharmaceutical and environmental compounds.* Nat Commun, 2015. **6**: p. 8089.

41. Hong, S., et al., *Heptachlor induced mitochondria-mediated cell death via impairing electron transport chain complex III.* Biochem Biophys Res Commun, 2013. **437**(4): p. 632-6.

42. Hansen, M.E., I.N. Pessah, and F. Matsumura, *Heptachlor epoxide induces a non-capacitative type of Ca2+ entry and immediate early gene expression in mouse hepatoma cells.* Toxicology, 2006. **220**(2-3): p. 218-31.

43. Chuang, L.F. and R.Y. Chuang, *Heptachlor and the mitogen-activated protein kinase module in human lymphocytes.* Toxicology, 1998. **128**(1): p. 17-23.
